# Genome-Wide Association Mapping of Flowering and Ripening Periods in Apple

**DOI:** 10.3389/fpls.2017.01923

**Published:** 2017-11-10

**Authors:** Jorge Urrestarazu, Hélène Muranty, Caroline Denancé, Diane Leforestier, Elisa Ravon, Arnaud Guyader, Rémi Guisnel, Laurence Feugey, Sébastien Aubourg, Jean-Marc Celton, Nicolas Daccord, Luca Dondini, Roberto Gregori, Marc Lateur, Patrick Houben, Matthew Ordidge, Frantisek Paprstein, Jiri Sedlak, Hilde Nybom, Larisa Garkava-Gustavsson, Michela Troggio, Luca Bianco, Riccardo Velasco, Charles Poncet, Anthony Théron, Shigeki Moriya, Marco C. A. M. Bink, François Laurens, Stefano Tartarini, Charles-Eric Durel

**Affiliations:** ^1^Institut de Recherche en Horticulture et Semences UMR 1345, INRA, SFR 4207 QUASAV, Beaucouzé, France; ^2^Department of Agricultural Sciences, University of Bologna, Bologna, Italy; ^3^Department of Agricultural Sciences, Public University of Navarre, Pamplona, Spain; ^4^Plant Breeding and Biodiversity, Centre Wallon de Recherches Agronomiques, Gembloux, Belgium; ^5^School of Agriculture, Policy and Development, University of Reading, Reading, United Kingdom; ^6^Research and Breeding Institute of Pomology Holovousy Ltd., Horice, Czechia; ^7^Department of Plant Breeding, Swedish University of Agricultural Sciences, Kristianstad, Sweden; ^8^Department of Plant Breeding, Swedish University of Agricultural Sciences, Alnarp, Sweden; ^9^Fondazione Edmund Mach, San Michele all'Adige, Italy; ^10^Plateforme Gentyane, INRA, UMR 1095 Genetics, Diversity and Ecophysiology of Cereals, Clermont-Ferrand, France; ^11^Apple Research Station, Institute of Fruit Tree and Tea Science, National Agriculture and Food Research Organization (NARO), Morioka, Japan; ^12^Wageningen UR, Biometris, Wageningen, Netherlands; ^13^Hendrix Genetics, Boxmeer, Netherlands

**Keywords:** adaptive traits, association genetics, germplasm collection, GWAS, *Malus* × *domestica* Borkh., microsynteny, quantitative trait loci, SNP

## Abstract

Deciphering the genetic control of flowering and ripening periods in apple is essential for breeding cultivars adapted to their growing environments. We implemented a large Genome-Wide Association Study (GWAS) at the European level using an association panel of 1,168 different apple genotypes distributed over six locations and phenotyped for these phenological traits. The panel was genotyped at a high-density of SNPs using the Axiom®Apple 480 K SNP array. We ran GWAS with a multi-locus mixed model (MLMM), which handles the putatively confounding effect of significant SNPs elsewhere on the genome. Genomic regions were further investigated to reveal candidate genes responsible for the phenotypic variation. At the whole population level, GWAS retained two SNPs as cofactors on chromosome 9 for flowering period, and six for ripening period (four on chromosome 3, one on chromosome 10 and one on chromosome 16) which, together accounted for 8.9 and 17.2% of the phenotypic variance, respectively. For both traits, SNPs in weak linkage disequilibrium were detected nearby, thus suggesting the existence of allelic heterogeneity. The geographic origins and relationships of apple cultivars accounted for large parts of the phenotypic variation. Variation in genotypic frequency of the SNPs associated with the two traits was connected to the geographic origin of the genotypes (grouped as North+East, West and South Europe), and indicated differential selection in different growing environments. Genes encoding transcription factors containing either NAC or MADS domains were identified as major candidates within the small confidence intervals computed for the associated genomic regions. A strong microsynteny between apple and peach was revealed in all the four confidence interval regions. This study shows how association genetics can unravel the genetic control of important horticultural traits in apple, as well as reduce the confidence intervals of the associated regions identified by linkage mapping approaches. Our findings can be used for the improvement of apple through marker-assisted breeding strategies that take advantage of the accumulating additive effects of the identified SNPs.

## Introduction

Flowering time in temperate plants is influenced by multiple environmental factors related to temperature and day length at different periods of the year (Wilczek et al., 2009; Cook et al., 2012; Abbott et al., 2015). For crop cultivation, floral timing is of utmost importance, because it is a major yield determinant (Jung et al., 2017). Temperate fruit trees use bud dormancy for adaption to seasonality (Campoy et al., 2011; Sánchez-Pérez et al., 2014; Ionescu et al., 2017): flowering occurs uniformly when the chilling and heating requirements associated with winter and spring have been fulfilled. In the context of global climate change, increasing temperatures tend to result in an acceleration of springtime phenological events (Hänninen and Tanino, 2011; Cook et al., 2012), with implications for both the risk of frost damage (Cannell and Smith, 1986; Vitasse et al., 2014) and the photosynthetic capacity of the trees (Ensminger et al., 2008). Moreover, this advance is responsible for several morphological disorders/abnormalities, including bud burst delay, low burst rate, irregular floral or leaf budbreak and poor fruit set (Erez, 2000; Celton et al., 2011; Dirlewanger et al., 2012; Abbott et al., 2015). Disruptions in synchronization of flowering may disturb pollination for self-incompatible cultivars, while modifications of fruit harvesting periods can cause problems with orchard management and fruit marketing (Dirlewanger et al., 2012). Breeding programs mainly focus on improvement of yield and fruit quality, but additional objectives like climate change adaptation receive increased attention. Genetic control of flowering and ripening periods plays a crucial role, since adaptation to different growing environments affects fruit quality (Chagné et al., 2014; Jung et al., 2017).

Flowering time is regulated by an intricate signaling network of multiple genes that integrates both endogenous and exogenous stimuli to induce flowering under the most favorable conditions (Boss et al., 2004; Amasino, 2005). Fruit ripening control involves coordinated regulation of many metabolic pathways (Johnston et al., 2009; Pirona et al., 2013; Chagné et al., 2014), resulting in the conversion of starch to sugars, reduced acidity, reduced flesh firmness, changes in color and an increase in aroma/flavor volatile compounds. Both traits are quantitatively inherited in most fruit tree species (Celton et al., 2011; Pirona et al., 2013; Castède et al., 2014; Chagné et al., 2014).

Association mapping exploits the linkage disequilibrium (LD) present among individuals from natural populations or germplasm collections to dissect the genetic basis of complex trait variation (Neale and Savolainen, 2004; Aranzana et al., 2005; Balding, 2006; Myles et al., 2009). Germplasm collections generally contain more genetic diversity than segregating progenies and, since association mapping exploits all the recombination events that have occurred in the evolutionary history of the association panel, a much higher mapping resolution is expected (Zhu et al., 2008; Myles et al., 2009; Ingvarsson and Street, 2011). In addition, the number of QTLs that can be mapped for a given phenotype is not limited to the segregation products in a specific cross, but rather by the number of QTLs underlying the trait and the degree to which the studied population captures the genetic species-wide diversity (Zhu et al., 2008; Yano et al., 2016). Association mapping has recently been applied to fruit tree species such as peach (Micheletti et al., 2015), apricot (Mariette et al., 2016) and apple (Leforestier et al., 2015; Migicovsky et al., 2016; Di Guardo et al., 2017; Farneti et al., 2017), especially after the release of high-density SNP arrays with uniform coverage of the whole genome (Chagné et al., 2012; Verde et al., 2012; Bianco et al., 2014) or Genotyping-by-Sequencing (Gardner et al., 2014).

The high density Axiom®Apple 480 K SNP array (Bianco et al., 2016) developed within the EU-FruitBreedomics project (Laurens et al., 2012; http://www.fruitbreedomics.com) was used for the first time in the present study to perform GWAS. Here, we focused on the analysis of the genetic control of flowering and ripening periods in a panel of almost 1,200 different genotypes distributed over six apple collections managed by six European institutes. We identified one genomic region associated with flowering period and three with ripening period. Co-variation between the genotypic frequencies at the significant SNPs and three major geographic groupings of genotypes was explored, and candidate genes were identified in the detected genomic regions. To our knowledge, this is the largest association study ever performed in a fruit tree species considering both population size and SNP marker density.

## Materials and methods

### Plant material

The association panel consisted of 1,168 different diploid apple genotypes corresponding to accessions preserved in six European germplasm collections (Table S1). The uniqueness of these genotypes was confirmed with SSR markers in a previous analysis (Urrestarazu et al., 2016). Some accessions corresponding to genotypes present at multiple locations were maintained in order to adjust phenotypic data between collections. Especially, ten standard genotypes (“Alkmene,” “Ananas Reinette,” “Discovery,” “Golden Delicious,” “Ingrid Marie,” “James Grieve,” “Jonathan,” “Reine des Reinettes” (= “King of the Pippins”), “Reinette de Champagne” and “Winter Banana”) were included from almost all collections. The association panel comprised mainly genotypes corresponding to old local/national cultivars, and the majority could be classified into three geographic groups according to their area of origin in Europe [North+East (141 different genotypes), South (148) and West (775)]; the remaining 104 corresponded to recent cultivars, germplasm originating from other worldwide regions, or were of unknown origin (Urrestarazu et al., 2016).

### Phenotypic data analysis

Phenotypic data for flowering and ripening periods were scored on an ordinal scale from 1 to 9, and consisted of both historical data in germplasm databases and of new data acquired in recent years (2012–2014) using the same scoring scales. Flowering period was assessed by recording dates of Fleckinger phenological flower stages F or F2 (Fleckinger, 1964), and then assigning a score on the ordinal scale by comparison to reference cultivars. Assessments for flowering period were performed over a period of 3–19 years except for NFC where only a single average value (assessed over 10 years) was available (Table 1). Ripening period was determined by observing pre-ripening drop of healthy fruits, ground- and over-color of fruits, taste of fruits and/or iodine starch index. It was recorded over 3–13 years (Table 1).

**Table 1:** Averages and ranges for the genotypic means for flowering and ripening periods.

**Population**	**Phenotypic assessments**			**Genotypic adjusted means**		
	**No. years (range)**	**data/cvr**.	**bs_h^2^**	**Mean**	***SD***	**Range**
**FLOWERING PERIOD**						
Whole population	29 (1985–2014)	5.45	0.82	4.79	1.15	1.73–9.24
INRA	4 (2009–2012)	3.00	0.88	5.58	1.45	2.56–9.52
UNIBO	6 (1987–1992)	7.60	0.84	5.26	0.99	2.57–7.81
CRA-W	19 (1985–2007)	4.90	0.88	3.97	1.03	1.25–7.25
RBIPH	13 (1995–2010)	5.00	0.85	4.42	0.83	3.03–8.61
NFC	1^a^	–	–	4.91	0.92	2.00–9.00
SLU	3 (2012–2014)	3.00	0.81	3.66	0.85	1.99–6.56
**RIPENING PERIOD**						
Whole population	22 (1987–2014)	5.37	0.95	5.43	2.05	0.54–9.95
INRA	10 (2002–2014)	4.86	0.95	6.89	1.77	1.62–9.26
UNIBO	13 (1987–2014)	7.83	0.96	6.51	1.87	0.98–9.19
CRA-W	10 (1987–2008)	4.37	0.87	4.90	1.15	1.12–8.38
RBIPH	5 (2006–2010)	5.07	0.92	5.00	1.56	1.00–7.60
NFC	3 (1999–2013)	2.93	0.87	5.94	1.77	2.00–8.33
SLU	3 (2012–2014)	2.91	0.98	3.75	1.44	1.00–7.00

a *A single average value was available at NFC, assessed over 10 years (different years according to the cultivars)*.

Genotypic means obtained for each genotype by adjusting for year and site effects were used as phenotypes for association analysis. When analyzing individual collections, the genotypic means were estimated using a linear model taking into account the year effect (Equation 1), while we considered the combined effect of site and year (Equation 2) for the whole analysis, i.e., all the collections were combined into a single analysis:

(1) $$\begin{array}{r} P_{ik}=\mu +Y_{i}+g_{k}+e_{ik} \end{array}$$

(2) $$\begin{array}{r} P_{ijk}=\mu +\left(Y_{i}\times S_{j}\right)+g_{k}+e_{ijk} \end{array}$$

where for (Equation 1), *P*_*ik*_ is the phenotypic value of the *k*th genotype in the *i*th year; μ is the mean value of the trait; *Y*_*i*_ is the fixed effect of the *i*th year on the trait; *g*_*k*_ is the random genotypic effect of genotype k; and *e*_*ik*_ is the residual term of the model. For (Equation 2), μ, *Y*_*i*_, and *g*_*k*_ have the same meanings as in (Equation 1); *P*_*ijk*_ refers to the phenotypic value of the *k*th genotype in the *i*th year in the *j*th site; *S*_*j*_ is the fixed effect of the *j*th site; and *e*_*ijk*_ is the residual term of the model. Heritability of genotypic means (*h*^2^, here called broad-sense heritability) was estimated for each individual collection as:

(3) $$\begin{array}{r} h^{2}=\;\frac{\sigma_{G}^{2}}{\sigma_{G}^{2}+\frac{\sigma_{\varepsilon }^{2}}{n}} \end{array}$$

where $\sigma_{G}^{2}$ is the variance of genotype effect, $\sigma_{\varepsilon }^{2}$ is the variance of the residual term, and *n* is the mean number of observations per genotype. These analyses were performed using “R” software (R Core Team, 2014), in particular the packages effects (Fox, 2003), lme4 (Bates and Sarkar, 2007) and FactoMineR (Lê et al., 2008).

### SNP genotyping

The 1,168 apple genotypes were genotyped with the Axiom®Apple 480 K array containing 487,249 SNPs evenly distributed over the 17 apple chromosomes (Bianco et al., 2016). Bianco et al. (2016) applied stringent filters that resulted in a set of 275,223 robust SNPs for GWAS. Further details on the development of the SNP array, genotyping process, and the filtering pipeline procedure can be found in Bianco et al. (2016). All presented results use the SNP positions on the latest version (v1.1) released for the apple genome based on the doubled haploid GDDH13 (hereafter, GDDH13 genome; Daccord et al., 2017; see also https://iris.angers.inra.fr/gddh13/ for the genome browser).

### Kinship, population structure and linkage disequilibrium estimates

GEMMA software (Zhou and Stephens, 2012) was used to estimate the standardized relatedness matrix (**K**) between the genotypes at the whole population level and within each collection. A Principal Component Analysis (PCA) of the SNP data was performed using PLINK (Purcell et al., 2007) and the ten largest Eigenvalues were used to control for population structure (**Q**). Matrix **Q** was constructed for the whole population as well as for each collection separately.

LD was studied between sets of SNPs spanning regions of 10 kb randomly sampled along the genome. These sets were obtained by a random choice of 50 contigs larger than 10 kb on each chromosome, followed by the selection of SNPs spanning a random region of 10 kb in each of these contigs. LD was estimated as squared allele frequency correlations (*r*^2^) and as *r*^2^ corrected for population structure and relatedness ($r_{\text{vs}}^{2}$) using the R-package LDcorSV (Mangin et al., 2012). In addition, local LD (*r*^2^) was assessed for chromosomal regions of 1 Mb surrounding the SNPs retained as cofactors in the GWAS (see next section for details) and displayed in LD maps and network plots using “LDheatmap” and “network” R-packages, respectively.

### Genome-wide association study (GWAS)

The GWAS method was applied both at the whole population level and for each collection independently. GWAS were conducted with correction for population structure (**Q**) and modeling phenotypic covariance with the kinship matrix (**K**) implemented in a modified version of the multi-locus mixed model (MLMM) proposed by Segura et al. (2012). The Extended Bayesian Information Criterion (EBIC, Chen and Chen, 2008) was used to select the model that best fitted our data. A genome-wide significance threshold was determined using a Bonferroni correction at 5%. MLMM uses a stepwise mixed-model regression with forward inclusion and backward elimination of SNPs re-estimating the variance components of the model at each step. MLMM divides the phenotypic variance into genetic variance (explained by structure, by kinship, and by SNPs included as cofactors in the model), and unexplained variance (residual variance), suggesting a natural stopping criterion (genetic variance = 0) for including cofactors, and allowing to estimate the explained and unexplained heritable variance for each trait. The causal-variant heritability tagged by all possible genotyped SNPs, was quantified for each trait at the step 0 of MLMM, i.e., when the structure, kinship and residual variances were estimated with no SNP included as cofactors in the model. The part of variance explained (PVE) by the significant SNP(s) as well as the part of variance due to population structure and due to kinship, were estimated at the optimal step of the MLMM (i.e., stopping criterion).

To establish 95% confidence intervals for the significant SNPs retained as cofactors in the whole population, we conducted a re-sampling approach as proposed by Hayes (2013). The full set of individuals with phenotypic data was randomly split into two subsets with equal size; this procedure was repeated 50 times for each trait, and then a GWAS was run on each subset as explained above. The standard error (*se*(*x*)) of the position of an underlying association was estimated as the median absolute deviation of the positions of the SNPs retained as cofactors on each chromosome over all subsets. Then, the 95% confidence interval was calculated as the position of the most significant SNPs retained as cofactors in the analysis of the whole population ±1.96 *se*(*x*).

### Effects of the SNPs identified as cofactors

The SNPs identified as cofactors were analyzed toward mode and size of allelic effects. The mode of gene action at each SNP was estimated for the whole population and (when possible) for each of the three geographic groups using the ratio of dominance (*d*) to additive (*a*) effects calculated from the mean of the genotypic means for each genotypic class. The dominance effect was calculated as the difference between the mean observed within the heterozygous class and the mean across both homozygous classes (*d* = G_AB_ – 0.5 (G_AA_ + G_BB_), where G_ij_ is the trait mean in the *ij*th genotypic class). To classify the mode, we used the following ranges, similar to Wegrzyn et al. (2010). No dominance was defined for small absolute values, i.e., |*d*/*a*| ≤ 0.50; partial or complete dominance was defined as values in the range 0.50 < |*d*/*a*| < 1.25; and over- or under-dominance pertained to values of |*d*/*a*| > 1.25.

To assess the joint effect of the different allelic combinations (i.e., genetic variants) defined by the SNPs identified for each trait, mean and statistical significance among the most frequent genetic variants were calculated by the Tukey-Kramer test (α = 0.05).

Dominance and epistatic effects among the identified SNPs were tested in a model including their additive effects with correction for population structure (**Q**) and modeling phenotypic covariance with the kinship matrix (**K**). Percentages of variance explained by additive plus dominance effects, and by additive plus dominance and epistatic effects were estimated in a hierarchical sequence, using a cumulative *R*^2^ metric.

### *In silico* candidate gene research

Chromosomal regions corresponding to approximate 95% confidence intervals for the position of SNPs retained as cofactors in the whole population for flowering and ripening periods were investigated for *in silico* candidate gene identification using GDDH13 genome (Daccord et al., 2017). The annotations of protein-coding and non-protein-coding genes of the regions of interest were identified using GDDH13 genome v1.1 browser (https://iris.angers.inra.fr/gddh13/). Annotations regarding the biochemical function of genes (mainly provided by InterproScan) were enriched by the biological functions inferred from the putative orthologs identified in *Arabidopsis thaliana, Solanum lycopersicum*, and *Prunus persica* genomes. Furthermore, structures of predicted genes and intergenic regions were systematically investigated to detect eventual mis/not-annotated genes and pseudogenes (stop codons and/or frameshifts in their CDS) in the regions of interest.

## Results

### Phenotypic variation

Large phenotypic variation was observed at both whole population and collection level (Table 1). In the individual collections, average flowering period varied in genotypic means from 3.66 to 5.58 (on the 1–9 scale), while average ripening period varied from 3.75 to 6.89. Heritability was consistently high (>0.80). The two traits were significantly correlated when calculated across all genotypes in the whole population (*r* = 0.44; *p*-value = 2.2e^−16^), see Figure S1.

**Figure 1:**
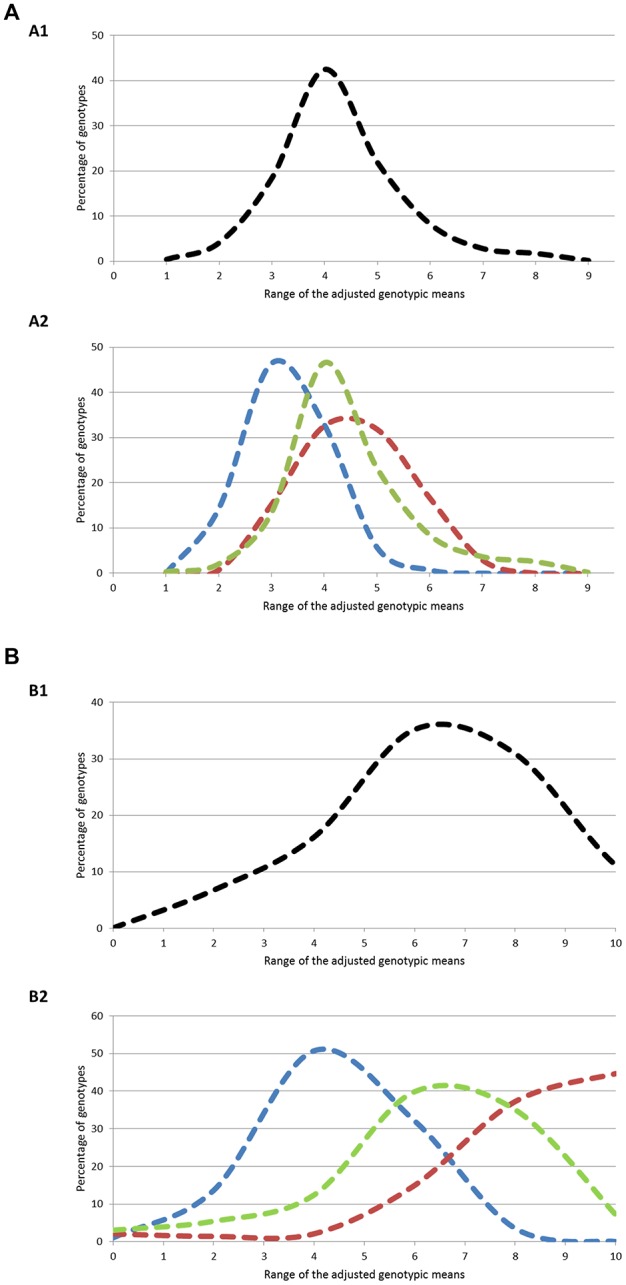
**(A)** Distribution of the genotypes according to ranges of genotypic means on flowering period at two different levels: (A1) Whole population; (A2) Geographic groups. The three geographic groups are depicted using the following color codes: Blue = North+East group; Green = West group; Red = South group. **(B)** Distribution of the genotypes according to ranges of genotypic means on ripening period at two different levels: (B1) Whole population; (B2) Geographic groups. The three geographic groups are depicted using the following color codes: Blue = North+East group; Green = West group; Red = South group.

The three geographical groups differed considerably for both traits (Figure 1). For flowering period, 94% of the genotypes had genotypic means between 2 and 5 (mean value = 3.77) in the North+East group, while 96 and 83% of the genotypes varied between 3 and 7 and between 3 and 6 for the South and West groups respectively, with almost identical mean values (South: 5.03; West: 4.99). For ripening period, phenotypic variation was even higher: 83% of the genotypes in the North+East group had a genotypic mean below 5 (mean value = 3.41), while 91% in the South group had values above 5 (mean value = 7.49). The West group showed an intermediate distribution (mean value = 5.48).

### Population structure and linkage disequilibrium

**Figure 2:**
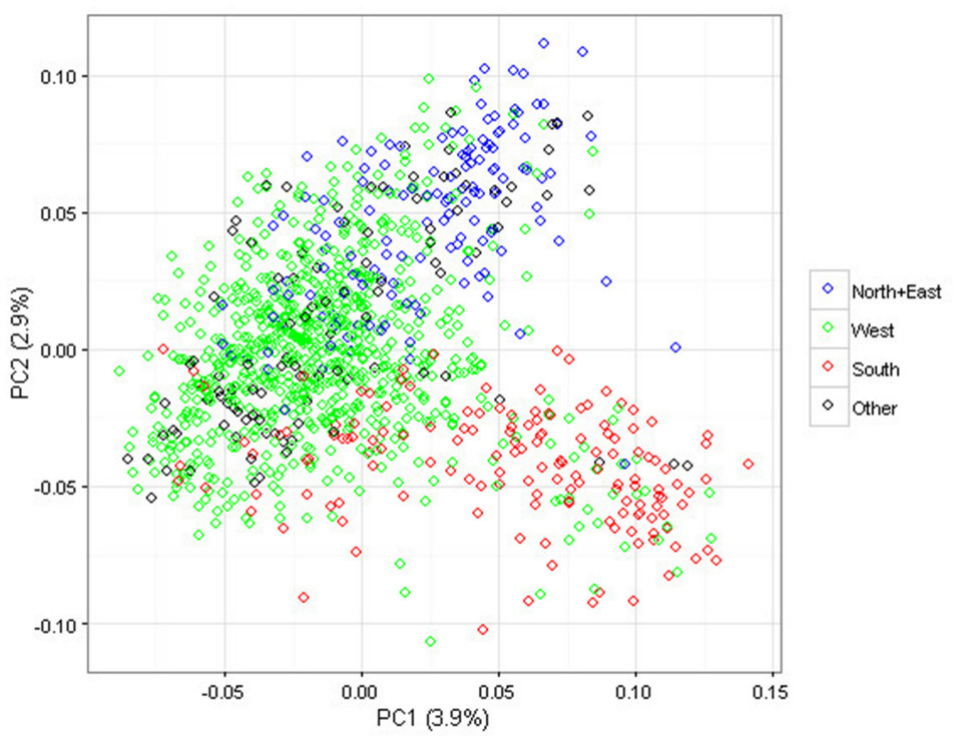
Scatter plot of the first two dimensions of the Principal Component Analysis (PCA) performed on the 1,168 apple genotypes based on 275,223 SNPs. The geographic groups are depicted using the following color codes: Blue = North+East group; Green = West group; Red = South group; Black = Other.

PCA was applied to summarize global genetic marker variation in the association panel: the ten largest Eigenvalues used to describe the whole population structure explained 17% of the overall variation. In Figure 2, the first two components of the PCA are represented. Genetic discrimination between the genotypes classified according to their geographic group of origin is visible in the bi-dimensional plot; genotypes from the North+East group are located in the upper part along the Y axis, while those from the West and South groups mostly occur on the left and right side along the X axis, respectively. The three groups North+East, West and South, explained 30 and 37% of the variation for the first two dimensions of the PCA but less than 6.5% for the next eight dimensions.

**Figure 3:**
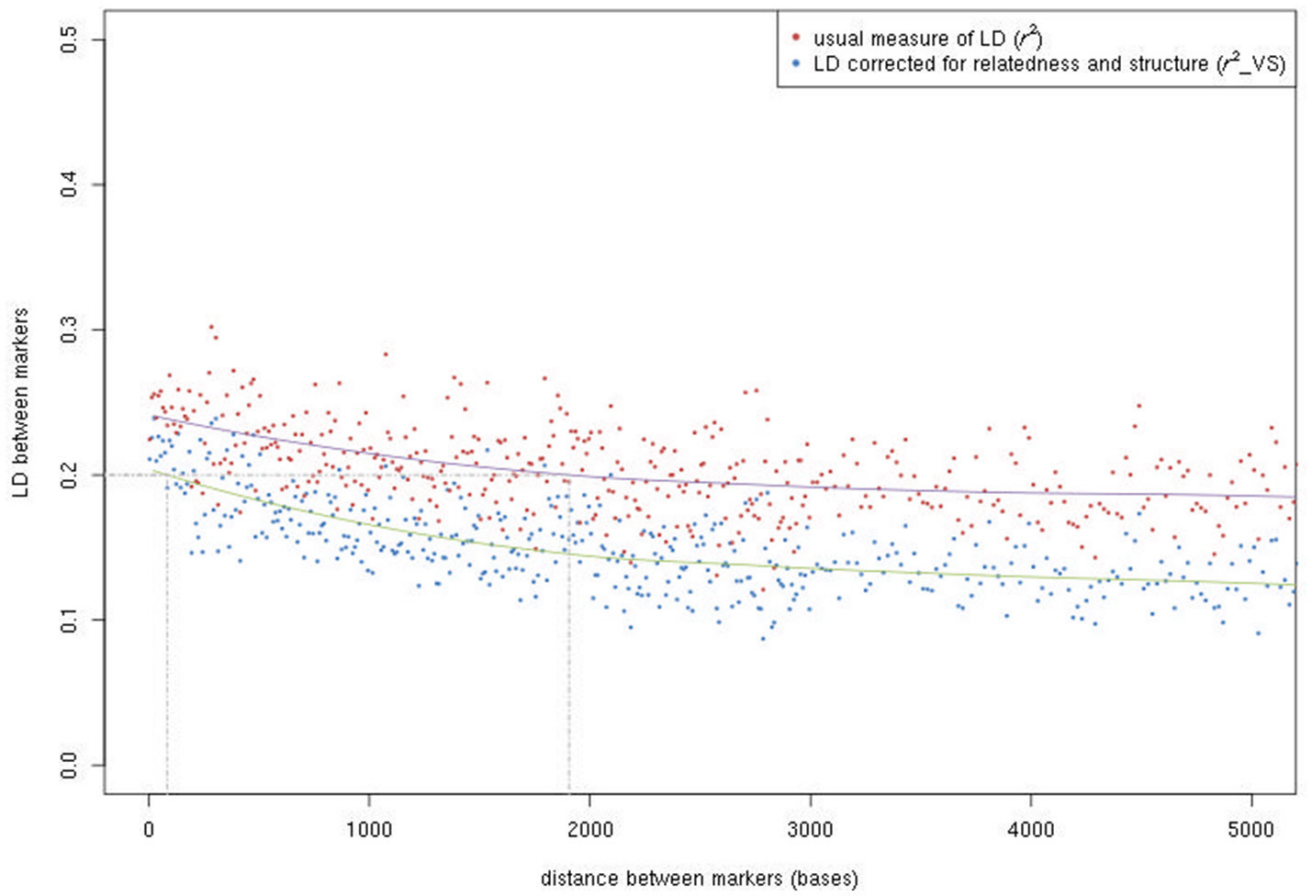
LD decay according to the physical distance between SNPs. Both the usual *r*^2^ and the *r*^2^ after correcting for relatedness and population structure ($r_{\text{vs}}^{2}$) are given.

LD (*r*^2^) was very variable in the SNP sets of 10 kb randomly sampled along the genome, spanning the entire range from absence to complete LD (Figure S2). The distribution of LD was highly asymmetric, with half of the marker pairs showing a *r*^2^ value below 0.1 ($r_{\text{vs}}^{2}$ value below 0.07 when corrected for relatedness and population structure). LD decay curves were very flat (Figure 3); mean *r*^2^ values were 0.24, 0.21, and 0.19 at 100 bp, 1 kb, and 5 kb, respectively, while mean $r_{\text{vs}}^{2}$ values were 0.20, 0.17, and 0.13, respectively. Half of the adjacent marker pairs occurred within 587 bp, while 90% occurred within 4,975 bp. To estimate LD between a causal variant in the middle of a marker interval and its flanking markers, mean *r*^2^ values for marker pairs at half these distances (i.e., 293.5 and 2,487.5 bp) were computed: in the whole population, mean *r*^2^ values were 0.23 and 0.19 without correction, and 0.19 and 0.14 with correction.

### Genome-wide association study (GWAS) without cofactor inclusion

#### Flowering period

Using a single-locus mixed model with control for population structure and relatedness, 50 SNPs were significantly associated with flowering period for the whole population (Table S2). In a quantile-quantile (Q-Q) plot (not shown), close adherence was found between the observed and expected -log10(*p*) values till around 3, indicating that the significant SNPs are unlikely to be biased by population structure and relatedness. A strong association signal was found on chromosome 9 (49 SNPs) with a Bonferroni correction threshold of 5% (–log10(*p*) > 6.74). The remaining SNP was located on the fictive chromosome 0 containing all unassigned scaffolds. The SNPs located on chromosome 9 spanned a distance of 3.24 Mb (265,164–3,509,888 bp).

Analyses of the individual collections revealed significant associations for flowering period only at INRA (29 SNPs), NFC (2 SNPs) and RBIPH (1 SNP) (Table S2). Twenty-one of these SNPs, all on chromosome 9, were also significant for the whole population. Of the 11 SNPs identified only in individual collections, nine were located on chromosome 9, one on chromosome 4 (RBIPH), and one on chromosome 11 (NFC).

#### Ripening period

For ripening period, 82 SNPs exhibited a significant association for the whole population with adjustment for population structure and relatedness (Table S3). The Q-Q plot (not shown) was similar to the previous one. Most SNPs (70) were located on chromosome 3, spanning a distance of 2.05 Mb (29,196,200–31,243,065 bp). Nine SNPs were located on chromosome 16 and spanned a distance of 274.3 kb (9,032,064–9,306,332 bp), while three SNPs could not be mapped.

When GWAS was performed for each collection separately, numbers of significant SNPs were 38, 12, and 8 for NFC, INRA and SLU, respectively, two for both RBIPH and UNIBO, and only one for CRA-W (Table S3). Thirty-one of the 43 SNPs identified in the analyses of individual collections, showed a significant association also in the whole population. When analyses were carried out at collection-scale, all the identified SNPs were located on chromosome 3, except for three that were unmapped; none of the SNPs located on chromosome 16 with a significant association in the whole population, were identified in the GWAS of the individual collections.

### Genome-wide association study (GWAS) using SNPs as cofactors

To further dissect the signal from the chromosomal regions containing the sets of significant SNPs for each trait, we performed a GWAS with MLMM using SNPs as cofactors (Segura et al., 2012). MLMM handles the putatively confounding effect of significant SNPs elsewhere on the genome, which considerably outperforms the existing single-locus mixed models by reducing the number of significantly associated SNPs rather than the number of peaks (Sauvage et al., 2014). The part of variance explained by structure and kinship estimated at step 0 of MLMM (i.e., when no SNP were included as cofactors in the model) was 0.78 for flowering period and 0.84 for ripening period (Table 2).

**Table 2:** Summary of trait associations at the optimal models according to the EBIC criterion.

**Population**	**No. cultivars**	**Model 0 PVE by (structure + kinship)**	**Optimum model**			
			**PVE by (structure + cofactors + kinship)**	**No. associations without cofactors**	**No. significant cofactors**	**PVE by cofactors**
**FLOWERING PERIOD**						
Whole population	1,126	0.78	0.75	50	2	0.09
INRA	251	0.93	0.90	29	1	0.13
UNIBO	166	0.74	0.74	0	0	0.00
CRA-W	221	0.72	0.72	0	0	0.00
RBIPH	177	0.79	0.58	1	2	0.27
NFC	288	0.78	0.77	2	4	0.33
SLU	159	0.80	0.80	0	0	0.00
**RIPENING PERIOD**						
Whole population	1,149	0.84	0.85	82	6	0.17
INRA	260	0.84	0.84	12	1	0.13
UNIBO	178	0.88	0.86	2	1	0.16
CRA-W	217	0.70	0.65	1	1	0.12
RBIPH	176	0.80	0.78	2	2	0.18
NFC	293	0.97	0.92	38	1	0.22
SLU	160	0.94	0.89	8	4	0.28

*Part of Variance Explained (PVE) by population structure, cofactors and kinship, the number of associations without cofactors, the number of significant cofactors, the PVE by cofactors, and the ratio PVE by kinship/PVE by cofactors and kinship are showed. Data obtained for individual collections and the whole populations are provided*.

#### Flowering period

**Figure 4:**
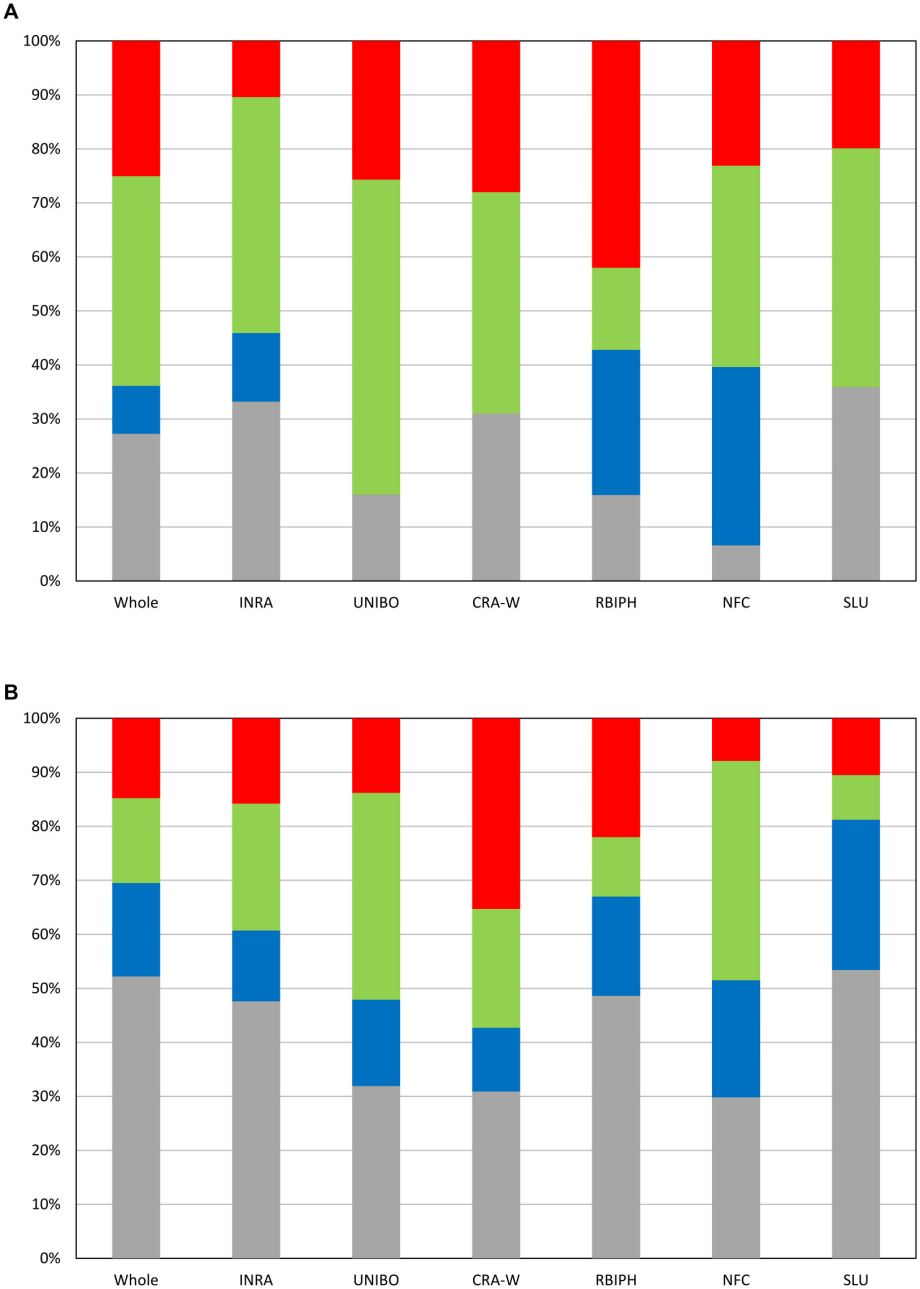
Partition of variance at the optimal models according to EBIC for the whole population and the six individual collections for flowering period **(A)** and ripening period **(B)**. Gray: part of variance explained by structure; Blue: part of variance explained by SNPs retained as cofactors; Green: part of variance explained by kinship; Red: residual variance.

The optimal MLMM according to the EBIC criterion for the whole population retained two SNPs for flowering (FB_AFFY_0496090 “SNP.9-1” and FB_AFFY_0495650 “SNP.9-2”), both located on chromosome 9, only 27 kb apart (Table 3). These two SNPs were significantly associated with flowering period also in the initial analysis based on a single-locus mixed model (Table S2). With this optimal model, 8.9% of the whole phenotypic variance was explained by the pair of SNPs retained, 27.3% corresponded to the underlying population structure of the association panel, and 38.6% was associated with kinship (Figure 4A). In the re-sampling analysis conducted for estimating an approximate 95% confidence interval, the number of cofactors retained in the model was one in 76 subsets, two in 23 subsets and three in one subset (Table S4). SNP.9-1 and SNP.9-2 were selected as cofactors in 35 and 38 subsets, respectively, while other SNPs from the same chromosome were selected as cofactors in 45 subsets, among which FB_AFFY_4941692 (“SNP.9-5”) was selected in 25 subsets (Table S4). For this region, the length of a 95% confidence interval was estimated at 157 kb.

**Table 3:** Summary of associations identified by Multi-Locus Mixed Model (MLMM) at the optimal models according to the EBIC criterion for flowering and ripening periods in the whole population and in the six individual collections.

**Population**	**SNP code**	**SNP short name**	**No. Cofactor^a^**	**Location of the SNPs**		**Alleles**	***p*-value**	**MAF**
				**Chromosome**	**Position**			
**FLOWERING PERIOD**								
Whole population	FB_AFFY_0496090	SNP.9-1	1	9	530,386	**G**/T^b^	1.33E-08	0.11
Whole population	FB_AFFY_0495650	SNP.9-2	2	9	557,419	**A**/G	6.81E-08	0.13
INRA	FB_AFFY_0495650	SNP.9-2	1	9	557,419	**A**/G	1.06E-12	0.18
RBIPH	FB_AFFY_6830175	SNP.4-1	1	4	968,334	C/**T**	3.16E-09	0.01
RBIPH	FB_AFFY_1629518	SNP.9-3	2	9	925,476	A/**G**	8.01E-08	0.14
NFC	FB_AFFY_6873601	SNP.4-2	4	4	7,719,622	A/**G**	3.94E-07	0.24
NFC	FB_AFFY_7355751	SNP.9-4	2	9	1,938,744	**C**/T	3.15E-08	0.11
NFC	FB_AFFY_2782466	SNP.11-1	1	11	12,422,656	A/**C**	8.30E-09	0.02
NFC	FB_AFFY_9818101	SNP.12-1	3	12	14,536,815	C/**T**	5.35E-08	0.15
**RIPENING PERIOD**								
Whole population	FB_AFFY_6730867	SNP.3-3	4	3	30,430,113	**A**/G^c^	6.76E-15	0.10
Whole population	FB_AFFY_7541229	SNP.3-4	5	3	30,465,002	C/**T**	8.51E-10	0.09
Whole population	FB_AFFY_4981462	SNP.3-6	2	3	30,700,183	C/**T**	4.39E-19	0.18
Whole population	FB_AFFY_1209620	SNP.3-7	1	3	30,726,252	A/**G**	1.28E-13	0.41
Whole population	FB_AFFY_3795860	SNP.10-1	6	10	38,390,484	**A**/G	1.76E-08	0.23
Whole population	FB_AFFY_6370928	SNP.16-1	3	16	9,146,297	C/**T**	5.16E-12	0.14
INRA	FB_AFFY_1253936	SNP.3-5	1	3	30,590,166	A/**C**	3.03E-14	0.08
UNIBO	FB_AFFY_1253936	SNP.3-5	1	3	30,590,166	A/**C**	6.75E-09	0.06
CRA-W	FB_AFFY_4741632	SNP.3-2	1	3	30,318,639	**A**/G	6.71E-08	0.11
RBIPH	FB_AFFY_4981462	SNP.3-6	1	3	30,700,183	C/**T**	3.60E-10	0.20
RBIPH	FB_AFFY_4836781	SNP.15-1	2	15	10,377,731	**C**/T	3.18E-08	0.37
NFC	FB_AFFY_4981462	SNP.3-6	1	3	30,700,183	C/**T**	1.37E-18	0.14
SLU	FB_AFFY_0899559	SNP.3-1	4	3	24,220,838	**A**/G	7.21E-07	0.10
SLU	FB_AFFY_1209620	SNP.3-7	1	3	30,726,252	A/**G**	7.51E-15	0.33^d^
SLU	FB_AFFY_6239519	SNP.13-1	3	13	1,889,560	**G**/T	1.41E-07	0.24
SLU	FB_AFFY_3879540	SNP.16-2	2	16	10,298,660	**G**/T	2.34E-07	0.27

a *Order of inclusion of the SNPs at the optimal model in MLMM according to the EBIC criterion*.

b *The allele associated with an early flowering period is highlighted in bold. The alternative allele is thus associated with a late flowering period*.

c *The allele associated with an early ripening period is highlighted in bold. The alternative allele is thus associated with a late ripening period*.

d *The allele found in SLU with the lowest frequency was the opposite to the one that appeared in the lowest frequency in the whole population and the other five individual collections*.

GWAS of each single collection retained one, two and four SNPs (Tables 2, 3) for the collections of INRA, RBIPH, and NFC, respectively, but none for the collections of CRA-W, SLU, and UNIBO. The part of variance explained by the markers selected in the optimal models for each collection was 13% (INRA), 27% (RBIPH), and 33% (NFC) (Table 2; Figure 4A). One of the two SNPs identified in the MLMM analysis of the whole population, SNP.9-2, was also found for the INRA collection. Neither the two SNPs selected for the RBIPH collection (chromosomes 4 and 9) nor the four identified in the NFC collection (chromosomes 4, 9, 11, and 12) were retained in the analysis of the whole population.

#### Ripening period

The optimal model according to the EBIC criterion retained six SNPs for the whole population, four at the bottom of chromosome 3 (FB_AFFY_6730867, FB_AFFY_7541229, FB_AFFY_4981462, and FB_AFFY_1209620, denoted as “SNP.3-3,” “SNP.3-4,” “SNP.3-6,” and “SNP.3-7”), one at the bottom of chromosome 10 (FB_AFFY_3795860 “SNP.10-1”), and another on the top of chromosome 16 (FB_AFFY_6370928 “SNP.16-1”) (Table 3). The four SNPs identified on chromosome 3 were clustered two by two with a distance of only 35 and 26 kb within each cluster and a distance of about 296 kb between clusters. The SNP retained on chromosome 10 (SNP.10-1) and one of the four retained on chromosome 3 (SNP.3-3) did not show a significant association with ripening period in the analysis based on a single-locus mixed model (Table S3). Altogether, the six SNPs explained 17.2% of the phenotypic variation, whereas population structure and kinship explained 52.2 and 15.7%, respectively (Figure 4B). When estimating the approximate 95% confidence interval with a re-sampling analysis, the number of cofactors retained varied between one and five, and between two and four in 94 of the 100 subsets (Table S5). SNP.3-6 and SNP.3-7, selected as the two first cofactors in the whole collection, were selected in 55 and 85 subsets, respectively. Other SNPs from chromosome 3 were selected in 59 subsets (Table S5). The length of a 95% confidence interval was estimated at only 152 kb for chromosome 3, but 1.39 Mb for chromosome 10, and 426 kb for chromosome 16.

The optimal model for the analysis of each individual collection retained at least one SNP per collection (Tables 2, 3): four in the SLU collection, two in the RBIPH collection, and one each in the remaining collections. Part of variance explained by the markers identified for each collection, ranged from 12% (CRA-W) to 28% (SLU) (Table 2; Figure 4B), with an average of 18%. Two out of the six SNPs retained in the whole population were identified also in some of the individual collections, i.e., SNP.3-6 in NFC and RBIPH, and SNP.3-7 in SLU, both of them belonging to the same lower cluster previously defined on chromosome 3. For INRA and UNIBO the same single SNP (FB_AFFY_1253936 “SNP.3-5”) on chromosome 3 was selected by MLMM and was located in between the two previously identified SNP clusters. In brief, the analyses of the individual collections identified six SNPs additional to the six ones identified in the whole population, three on chromosome 3, and one on each of chromosomes 13, 15, and 16.

For each trait, Manhattan plots obtained with the single-locus mixed model and the multi-locus mixed model for the whole population and for the individual collections, are shown in Figures S3, S4.

### Linkage disequilibrium among SNPs identified as cofactors

Pairwise LD was assessed to test the independence of SNPs identified as cofactors for each trait in the whole population as well as in each geographic group. For flowering period, low LD (*r*^2^ = 0.27; $r_{\text{vs}}^{2}$ = 0.12) was detected in the whole population between the two SNPs associated with the trait despite being located only 27 kb apart (Table 4A). Analysis of the results at the geographic-group level found almost complete equilibrium between these two SNPs in the North+East and South groups (*r*^2^ = 4E^−04^ and 0.06, respectively), while LD was much higher (*r*^2^ = 0.41; $r_{\text{vs}}^{2}$ = 0.22) in the West group. For ripening period, variable LD values were found among the four SNPs identified as cofactors located on chromosome 3 (Table 4B). Intermediate to high *r*^2^ values were found between SNP.3-3, SNP.3-4, and SNP.3-6, whereas low values were observed between these three SNPs and SNP.3-7. In the North+East and West groups, *r*^2^ values between these four SNPs were quite similar to those found in the whole population. By contrast, very low *r*^2^ were found for the South group, except for the pair SNP.3-3/SNP.3-6 (*r*^2^ = 0.43).

**Table 4:** **(A)** Pairwise LD between the two SNPs associated with flowering period in the whole population (A1) and in the three geographic groups: North+East (A2), West (A3), and South (A4). **(B)** Pairwise LD between the four SNPs associated with ripening period on chromosome 3 in the whole population (B1) and the three geographic groups: North+East (B2), West (B3), and South (B4).

**A**				**B**					
**(A1) Whole population**				**(B1) Whole population**					
SNPs as cofactors	SNP.9-1	SNP.9-2	MAF	SNPs as cofactors	SNP.3-3	SNP.3-4	SNP.3-6	SNP.3-7	MAF
SNP.9-1	1.00	0.12	0.11	SNP.3-3	1.00	0.55	0.31	0.02	0.10
SNP.9-2	0.27	1.00	0.13	SNP.3-4	0.71	1.00	0.27	0.06	0.09
				SNP.3-6	0.56	0.54	1.00	0.22	0.18
**(A2) North**+**East group**				SNP.3-7	0.11	0.06	0.22	1.00	0.41
SNPs as cofactors	SNP.9-1	SNP.9-2	MAF						
SNP.9-1	1.00	2.0E-03	0.09	**(B2) North**+**East group**					
SNP.9-2	4.2E-04	1.00	0.11	SNPs as cofactors	SNP.3-3	SNP.3-4	SNP.3-6	SNP.3-7^a^	MAF
				SNP.3-3	1.00	0.79	0.36	0.09	0.28
**(A3) West group**				SNP.3-4	0.83	1.00	0.32	0.11	0.27
SNPs as cofactors	SNP.9-1	SNP.9-2	MAF	SNP.3-6	0.48	0.45	1.00	0.33	0.45
SNP.9-1	1.00	0.22	0.13	SNP.3-7^a^	0.16	0.21	0.28	1.00	0.33 (0.67)^a^
SNP.9-2	0.41	1.00	0.14						
				**(B3) West group**					
**(A4) South group**				SNPs as cofactors	SNP.3-3	SNP.3-4	SNP.3-6	SNP.3-7	MAF
SNPs as cofactors	SNP.9-1	SNP.9-2	MAF	SNP.3-3	1.00	0.69	0.34	0.04	0.06
SNP.9-1	1.00	0.07	0.09	SNP.3-4	0.77	1.00	0.37	0.07	0.06
SNP.9-2	0.06	1.00	0.05	SNP.3-6	0.49	0.55	1.00	0.21	0.12
				SNP.3-7	0.09	0.12	0.21	1.00	0.39
				**(B4) South group**					
				SNPs as cofactors	SNP.3-3	SNP.3-4	SNP.3-6	SNP.3-7	MAF
				SNP.3-3	1.00	0.02	0.41	0.02	0.11
				SNP.3-4	0.01	1.00	0.04	4.7E-03	0.01
				SNP.3-6	0.43	0.07	1.00	0.03	0.13
				SNP.3-7	0.03	0.02	0.04	1.00	0.17

*Values below the diagonal line refer to the usual r^2^ and above the diagonal line refer to ${\text{r}}_{\text{vs}}^{2}$ (i.e., with correction for relatedness and population structure) MAF of the SNPs are given for the whole population and the three geographic groups*.

a The allele found in the North-East group with the lowest frequency at the SNP.3-7 was the opposite to the one that appeared in the lowest frequency in the whole population and the other two geographic groups.

*Pairwise LD between SNPs are highlighted using the following color scale: red (r^2^ = 1) and yellow (r^2^ = 0)*.

LD interconnections between the eight SNPs retained as cofactors and other SNPs residing within their surrounding regions of 1 Mb (Figure S6) showed that SNP.16-1 exhibited the highest number of connections with other SNPs at *r*^2^ > 0.70, i.e., 53 SNPs delineating a region of 763 kb (Table S6). None or only a few (maximum 16) SNPs in the neighborhood of the remaining seven retained SNPs were linked with them at *r*^2^ > 0.70. Accordingly, none of the triangular LD heat maps for the above mentioned regions showed a LD spatial pattern suggesting that they are organized in blocks of moderate/high LD (Figure S5). Conversely, networks of moderate LD (*r*^2^~0.5–0.6) were more frequently observed for the above mentioned regions (i.e., five regions with more than 40 connected SNPs).

### Allele frequencies, effects and genetic variants for the SNPs identified as cofactors

For each of the eight SNPs identified as cofactors in the analysis of the whole population, the minor (i.e., less frequent) allele remained the same across the individual collections, the three geographic groups and the whole population, except for two cases: SLU collection and North+East group for SNP.3-7 associated with ripening period (Tables 3, 4A,B). In the two latter cases, the allele of SNP.3-7 with the lowest frequency was the alternate one to that in the other five collections and two geographic groups thus exhibiting a strong shift in the frequency of the G allele associated to early ripening period. Large differences between geographic groups were also observed for the minor allele frequencies (MAF) of the four SNPs associated with ripening period located on chromosome 3 (Table 4B), again indicating a North-South gradient.

The phenotypic effects of the two SNPs identified for flowering period indicated a strong mean difference (>1.9) between genotypic means for genotypes homozygous for the alternative alleles (Table S7; Figure S7). Dominance effects and epistatic interaction effects were significant, despite explaining a very small part of the variance (Table 5; Table S7). For ripening period, an even higher mean difference (frequently >2.5) was observed between the genotypic means of alternative homozygous genotypes for the four SNPs on chromosome 3 (Table S8; Figure S8), while less variation was found for SNP.10-1 and SNP.16-1. Variation in genotypic frequencies at each SNP was again very pronounced between geographic groups (Figure S8). Globally, dominance effects and epistatic interaction effects between the six SNPs were not significant, except some partial dominance occasionally observed for SNP.3-3, SNP.3-4, and SNP.10-1 (Table 5; Table S8).

**Table 5:** Test of dominance and epistatic effects among the SNPs selected as cofactors in the GWAS of the whole population for flowering and ripening periods.

**Trait**	**Effects**	**d.f**.	***F*-test**	***p*-value**	**PVE (%)**
Flowering period	Additive	2	79.8	4.3E-33	9.1
	Dominance	2	5.9	2.7E-03	0.7
	Epistatic	4	9.0	3.7E-07	2.0
Ripening period	Additive	6	106.6	3.4E-106	17.4
	Dominance	6	1.1	3.6E-01	0.2
	Dominance of SNP.3-4	1	4.8	2.8E-02	0.1
	Dominance of SNP.3-6	1	4.5	3.3E-02	0.1
	Epistatic	41^a^	1.2	2.3E-01	1.3

a *Some combinations of SNP genotypes did not exist in the whole population, which reduced the df for all interactions between 6 SNPs*.

The joint effect associated with the two SNPs identified for flowering period was assessed by comparing the average values for genotypes with different combinations of alleles in the whole population. Among the five genetic variants with a frequency above 1% (Table 6), variants 1 and 5 combining, respectively, the two alleles associated with early (GG/AA) and late (TT/GG) flowering at a homozygous stage differed on average by 3.73 corresponding to 3.24 σ (in standard-deviation units). The double heterozygous variant 3 (GT/GA) exhibited an intermediate value. For the four SNPs identified on chromosome 3 for ripening period, only 26 combinations out of the 81 potential variants were observed, 10 of which accounted for ~95% of the association panel (Table 6). The genetic variants accumulating homozygous alleles associated to early ripening period (variant 10: AA/TT/TT/GG) or late ripening period (variant 1: GG/CC/CC/AA) differed by 4.63 on average corresponding to 2.25 σ. Out of the 397 genotypes belonging to variant 1, only 4.3% belonged to the North+East group, while 69.7 and 19.7% belonged to the West and South groups, respectively, representing 12, 35.7, and 52.7% of the total genotypes from North+East, West, and South groups, respectively. By contrast, the infrequent variant 10 (~2%) was common in the North+East group (52.2%) but more scarce (17.4%) and totally absent in the West and South groups, respectively. Multiple comparisons indicated no significant differences between variants 1 and 8, between variants 4, 5, 6, and 7, or between variants 9 and 10 (Table 6).

**Table 6:** Joint effect of the two SNPs associated with flowering period in the whole population and of the ten most frequent genetic variants defined by the four SNPs on chromosome 3 associated with ripening period in the whole population.

**Genetic variant**	**Genotypes at SNPs^a^^,^^b^**	**N°cultivars**	**Frequency**	**Mean**	**Median**	**SD**	**Min**	**Max**	**Tukey groups**
**FLOWERING PERIOD**									
Variant 1	**GG**/**AA**	760	0.67	4.65	4.73	0.95	1.73	8.87	a
Variant 2	**G**T/G**A**	126	0.11	5.83	5.73	1.32	2.73	8.88	b
Variant 3	**GG**/G**A**	121	0.11	4.43	4.47	1.13	1.73	8.24	a
Variant 4	**G**T/**AA**	89	0.08	4.49	4.67	1.00	2.34	7.37	a
Variant 5	TT/GG	11	0.01	8.38	7.82	1.08	5.73	9.24	c
**RIPENING PERIOD**									
Variant 1	GG/CC/CC/AA	397	0.35	6.75	6.80	1.43	2.21	9.84	a
Variant 2	GG/CC/CC/A**G**	336	0.29	5.69	5.63	1.35	0.88	9.50	b
Variant 3	GG/CC/CC/**GG**	73	0.06	4.72	4.81	1.61	1.21	8.48	c
Variant 4	GG/CC/C**T/**A**G**	61	0.05	3.75	3.74	1.73	0.88	8.77	d
Variant 5	**A**G/C**T**/C**T**/A**G**	59	0.05	3.75	3.85	1.35	1.21	7.15	d
Variant 6	**A**G/C**T**/C**T**/**GG**	44	0.04	2.89	2.54	1.32	0.54	6.85	d
Variant 7	GG/CC/C**T**/**GG**	39	0.03	3.49	3.21	1.61	0.55	6.66	d
Variant 8	**A**G/CC/C**T**/AA	29	0.03	7.44	7.67	1.51	4.82	9.80	a
Variant 9	**A**G/C**T**/**TT**/**GG**	28	0.02	2.01	2.09	0.62	1.16	4.14	e
Variant 10	**AA**/**TT**/**TT**/**GG**	23	0.02	2.11	2.21	1.10	0.54	4.42	e

a *The allele associated with an early flowering period is highlighted in bold; order of SNPs is as follows: SNP.9-1/SNP.9-2*.

b *The allele associated with an early ripening period is highlighted in bold; order of SNPs is as follows: SNP.3-3/SNP.3-4/SNP.3-6/SNP.3-7*.

### Candidate gene identification

For flowering period, we considered the interval 451,830–635,974 bp (i.e., 184 kb) on chromosome 9, corresponding to the fusion of the 95% confidence intervals of the two SNPs selected as cofactors. In this interval, we found 28 gene models (Table S9) including putative transcription factors containing e.g., a NAM/NAC (MD09G1006400), a WRKY (MD09G1008800), a SBP (MD09G1008900) domain, and a putative glutaredoxin (MD09G1007400). In a second run, we also considered the 95% confidence interval covering SNP.9-5 which was selected in 25 subsets of the re-sampling analysis (Table S4) despite not being detected in the initial analysis. The corresponding interval 654,780–811,891 bp (i.e., 157 kb) was almost contiguous to the previous one, thus defining a wider region of ~360 kb (451,830–811,891 bp). Thirty-eight additional gene models were found in this enlarged interval (Table S9) including a putative SRF transcription factor containing a MADS- and a K-box (MD09G1009100), another putative SRF transcription factor (not detected by automatic annotation pipeline), and a gene model containing a SWIB/MDM2 domain (MD09G1011600).

For ripening period, we considered two intervals on chromosome 3, one corresponding to the fusion of the 95% confidence intervals of SNP.3-6 and SNP.3-7 which overlapped (30,624,429–30,802,006 bp, i.e., 178 kb), and the second for the confidence interval of SNP.3-3 and SNP.3-4 (30,354,359–30,540,756 bp, i.e., 186 kb). Only eleven gene models were found in the first interval and 24 in the second, with 6 additional gene models in between (Table S10). Two successive genes encoding a putative transcription factor containing a NAM/NAC domain (MD03G1222600 and MD03G1222700) were found in the very close vicinity of SNP.3-6 and SNP.3-7, both SNPs located in between the two genes. An Ultrapetala transcription factor (MD03G1220200) was found close to SNP.3-3, and a protein tyrosine kinase (MD03G1221300) close to SNP.3-4. On chromosome 10, we considered the 95% confidence interval 37,695,471–39,085,497 bp for SNP.10-1 and found 153 gene models (Table S11). Among them were four putative transcription factors, two of which contained a NAM/NAC domain (MD10G1288300 and MD10G1299900) while another two contained an Apetala-2 domain (MD10G1290400 and MD10G1290900). A carbohydrate phosphorylase putatively involved in starch metabolism was also identified (MD10G1289300). On chromosome 16, we considered a 95% confidence interval 8,933,453–9,359,141 bp for SNP.16-1 and found 38 gene models (Table S12). Together with two putative transcription factors encoding either a NAM/NAC domain or a TIFY domain (MD16G1125800 and MD16G1127400, respectively), we especially identified a gene model encoding an auxin responsive protein (MD16G1124300) and another gene model encoding a sugar bidirectional transporter (MD16G1125300).

A nearly perfect microsynteny (with some minor re-arrangement) was revealed between apple and peach in all the four confidence interval genomic regions estimated in our study, as shown by the numerous conserved homologs between the two species in those regions (Tables S9–S12).

## Discussion

### Genomic regions controlling variation in phenological traits

The SNPs retained as cofactors in the GWAS on the whole population defined one genomic region controlling flowering period and three controlling ripening period. Additional regions were identified when conducting GWAS for individual collections. The associations found accounted for varying levels of trait variation (0–33% for flowering period; 12–28% for ripening period) across the whole population and individual collections. We applied a conservative approach in identifying SNPs as cofactors for *p*-values below a defined threshold of Bonferroni correction at 5%. Implementation of those stringent parameters was essential to eliminate false positives, but have probably sacrificed some true associations with small effects.

The top of chromosome 9 was recently indicated as being involved in the genetic control of flowering or bud burst period (Celton et al., 2011; Allard et al., 2016). The regions pointed out in these contributions overlap with the confidence interval found in our study although the region indicated by Allard et al. (2016) is shifted slightly downstream since the very top of the chromosome was not mapped in their experiment. The regions indicated in these studies were, however, much larger than the confidence interval we report: Celton et al. (2011) examined a region of almost 16 cM corresponding to 4.04 Mb and comprising 983 gene models in the apple genome v1.0 of the Genome Database for *Rosaceae* (GDR, https://www.rosaceae.org/), whereas Allard et al. (2016) indicated a region of 10 cM corresponding to 1.8 Mb and comprising 622 gene models. The numerous recombination events accumulated in our association panel reduced the associated region to 184 kb with only 28 gene models in the GDDH13 genome. An extended interval of ~360 kb was nevertheless proposed to take into account the results of the re-sampling analysis, thus generating a final set of 66 candidate gene models. Interestingly, Trainin et al. (2016) identified a common haplotype on the top of chromosome 9 shared by a small subset of mostly Israeli apple cultivars adapted to low-chill conditions such as the well-known “Anna.” They defined an interval of about 1.7 Mb but suggested that the genetic factor/s responsible for early bud-break could be located in a region of about only 190 kb (between SNP-A6-2 and SNP-A4). Mapping these SNPs on the GDDH13 genome, we found the corresponding interval to be 730,978–923,844 bp, which overlaps the extended interval accounting for SNP.9-5 (451,830–811,891 bp). This co-localization raises the question of the allelic control of flowering period in that particular genomic region as described below.

Chromosomes 3, 10, and 16 have shown associations with ripening period in previous linkage mapping studies (Liebhard et al., 2003; Kenis et al., 2008; Chagné et al., 2014; Kunihisa et al., 2014). None of these studies attempted to define a confidence interval for the physical position of the reported QTLs, thus preventing an accurate comparison of the precision in QTL location between studies. Recently, Migicovsky et al. (2016) did not find any associations with ripening period on chromosomes 10 and 16 in a GWAS based on single-locus tests, but identified associations with two SNPs on chromosome 3 located within the coding region of NAC18.1 (GenBank ID: NM_001294055.1) which corresponds to a gene model (MD03G1222600) at position ~30,697,000 bp of GDDH13 genome. Interestingly, this position fits perfectly within the 95% confidence interval of SNP.3-6/SNP.3-7 (30,624,429–30,802,006 bp). Since this genomic region has been identified in various environments and genetic backgrounds, it therefore appears to potentially be a major factor in the genetic control of ripening period.

### GWAS on phenological traits suggests presence of allelic heterogeneity

For each trait, MLMM analysis for the whole population retained SNPs in weak LD despite being in close vicinity. Two SNPs retained as cofactors for flowering period on chromosome 9 were only 27 kb apart. Four SNPs retained for ripening period on chromosome 3 spanned a region of 296 kb, with two sub-regions spanning only 35 and 26 kb, respectively. Identification of multiple significant SNPs within or near a single gene may suggest either allelic heterogeneity or the presence of an untyped causal variant that requires multiple SNPs to be adequately tagged, or both (Atwell et al., 2010; Dickson et al., 2010; Segura et al., 2012). Allelic heterogeneity refers to the presence of more than two functional alleles of a given gene affecting a phenotypic trait (Wood et al., 2011). Indeed, the biallelic nature of SNPs reduces their ability to tag multiple alleles and explains the need for several SNPs to tag them. Also, maximizing the genetic variance in the association panel by including geographically distant accessions with both different and complex evolutionary histories is expected to improve resolution, but has the potential to introduce genetic heterogeneity (i.e., multiple causal variants with various dates of appearance and frequencies) which can generate false “synthetic” associations when only single-locus tests are used (Korte and Farlow, 2013). Fortunately, the MLMM approach is able to disentangle the contribution of genetic heterogeneity by including “competing” variants as cofactors within the mixed model setting and thus helps to discard false “synthetic” associations (Segura et al., 2012; Korte and Farlow, 2013). For flowering period, the two detected SNPs can either fit with allelic heterogeneity or untyped causal variant requesting more than one SNP. But more interestingly, the co-localization of our confidence interval with the small genomic region identified by Trainin et al. (2016) for the extreme phenotype of low-chilling requirement, opens the question of the local genomic architecture of this trait. Since bud-break and consequently flowering period of Israeli cultivars occur much earlier than in traditional European cultivars (Trainin et al., 2016), either two different polymorphic genes or a single gene with at least three alleles may be responsible for the co-location of detectable genotypic variation for flowering period and early bud-break. In the latter case, at least two alleles would control the genotypic difference we observed here for flowering period, and another more “extreme” allele would confer the early bud-break of Israeli cultivars. Alternatively, this extreme allele could be proposed as an epi-allele when considering epigenetic control (Ríos et al., 2014). For ripening period, a model including the two nearby genomic regions detected on chromosome 3 can also be proposed with the presence of two closely positioned genes (~300 kb apart), each with possible allelic heterogeneity. Such a complex pattern of association has never been highlighted before for flowering and ripening periods in apple. Nevertheless, additional genetic studies would be required to be certain about the multi-allelic and multi-genic architecture of the detected regions by using e.g., local haplotype sharing methods (Xu and Guan, 2014) provided that sufficient SNPs are available.

### Unexplained genetic variation may be accounted for by multiple factors

The limited number of detected genomic regions associated with the traits and the low/moderate amount of phenotypic variance accounted for by the retained SNPs suggests that several, if not many additional genomic regions are involved in the genetic control of these traits. Here, as with other GWAS, we were challenged by the so-called “missing heritability” syndrome (i.e., traits exhibiting both high heritability and tiny effect variants; Maher, 2008; Manolio et al., 2009; Visscher et al., 2010; Yang et al., 2010; Zuk et al., 2012). In our experiment, a significant proportion of the phenotypic variance not captured by the SNP cofactors could be explained by relatedness accounting for polygenic effects (15–58% for flowering period, 8–41% for ripening period) and population structure mostly accounting for genetic differentiation over geographic groups (7–36% for flowering period, 30–53% for ripening period). The large proportion of phenotypic variance under genetic control clearly indicates that additional genomic regions are still to be discovered. Interestingly, at the whole population level, the part of variance explained by relatedness for flowering period (39%) was more than twice the estimate for ripening period (16%), while the inverse was observed for the part of variance explained by structure (27% for flowering period, 52% for ripening period), thus indicating differential impact of relatedness and geographic structure on these two phenological traits.

Several factors may have hampered the detection of additional genomic regions. Genetic architecture consisting of many common variants with small effects and/or rare variants with large effects can reduce the statistical power of GWAS (Brachi et al., 2011; Gibson, 2011; Stranger et al., 2011; Korte and Farlow, 2013). The wide diversity in our association panel may have favored the inclusion of several rare variants with strong effects that could not be detected in the present study. The rapid LD decay and the LD pattern between causal variants and genotyped SNPs are two other limiting factors (Manolio et al., 2009; Visscher et al., 2010; Stranger et al., 2011). Despite the use of a high-density SNP array, it is possible that some genomic regions with causal variants were insufficiently covered by SNPs (i.e., null or incomplete LD), thus preventing detection of the corresponding variance. Denser genotyping may be required to find new associations given that both their effect and frequency are large enough to be detected by GWAS. Also, other factors may account for the unexplained genetic variation: (i) quality and precision of the phenotypic (historical) data (Myles et al., 2009; Migicovsky et al., 2016), (ii) genotype × environment (GxE) interactions, (iii) epistatic effects that were not systematically investigated in our experiment, or even, (iv) epigenetic variation.

### Population structure and geographic adaptation

Our association panel consisted mostly of local and/or old dessert apple cultivars selected as representative subsets by each institute. The phenotypic differences observed in the geographic-scale analyses (North+East, South and West groups) are probably explained by adaptive selection to different environments. Adaptive traits are frequently filtered by environmental gradients that coincide with patterns of population structure due to the differential fixation of alleles among groups of cultivars, following diversifying selection and/or genetic drift (Atwell et al., 2010; Brachi et al., 2011; Lasky et al., 2015; Nicolas et al., 2016). Despite genetic structure being weak at the whole population scale in our study (only 17% of the genotypic variation was explained by the ten largest Eigenvalues of the PCA), this structure explained a moderate (flowering period: 27%) or even high (ripening period: 52%) proportion of the phenotypic variance in GWAS. These results are in line with the phenotypic differences observed at a geographic scale, since the first two principal components were highly associated with geographic grouping (30 and 37%). A similar observation was made by Migicovsky et al. (2016). Differential selection together with genetic drift where the germplasm originated (North+East, West and South) may have favored or selected specific alleles or combinations of alleles in different geographic regions/environments. A good example is given by SNP.3-7, which was associated to ripening period with a frequency of 67% for its G allele in the North+East group but only 17% in the South group. Similarly, when considering the genetic variants combining the four SNPs retained on chromosome 3, variant 10 combining all earliness-associated SNP alleles at a homozygous state, was very common in accessions of the North+East group while totally absent in the South. In apple, harvest period is probably the trait with the strongest impact of geographical adaptation, since local weather conditions define the length of harvesting season.

### Putative functions of genes controlling phenotypic variation in apple flowering period

Gene models of particular interest were identified in the interval defined on chromosome 9, including a putative NAC gene (MD09G1006400). NAC-domain proteins are transcription factors involved in the genetic control of flowering time in *Arabidopsis* (Yoo et al., 2007), where two NAC proteins in association with a JMJ14 gene (a histone demethylase) apparently take part in flowering time regulation (Ning et al., 2015). In addition, a putative WRKY transcription factor was identified. This gene model (MD09G1008800, corresponding to MDP0000154734 in GDR) was cited by Trainin et al. (2016) as a putative candidate for early bud-break of Israeli apple cultivars. The WRKY gene family was recently proposed to play a role in dormancy regulation in peach (Chen et al., 2016). Based on RNAseq, MD09G1008800 transcription was detected mainly in apple roots and only slightly in fruits, thus limiting its potential role in flowering.

Three other candidate gene models are of special interest for the genetic control of flowering period. MD09G1009100 and another non-predicted gene model are similar to SRF transcription factors containing a MADS domain, putatively homologous to the *FLC* (*FLOWERING LOCUS C*) gene involved with the *FRIGIDA* gene in vernalization response of *Arabidopsis* (reviewed by Amasino and Michaels, 2010). MADS-box genes, such as the DAM (dormancy associated MADS-box) family members, were previously shown to be the master regulators of dormancy establishment and maintenance in *Prunus* and *Pyrus* species (Bielenberg et al., 2008; Ubi et al., 2010; Yamane et al., 2011; Saito et al., 2013; Sánchez-Pérez et al., 2014; Zhebentyayeva et al., 2014). Related DAM-like genes with dormancy-dependent expression have been identified in other perennial species such as leafy spurge (Horvath et al., 2008, 2010), raspberry (Mazzitelli et al., 2007), blackcurrant (Hedley et al., 2010), and kiwifruit (Wu et al., 2012). Also, MD09G0010600 is predicted as a SWIB/MDM2-domain containing gene, a member of a family of chromatin remodeling complexes that modify DNA accessibility by restructuring nucleosomes (Jerzmanowski, 2007). These three genes (MDP0000167381/MDP0000126259, MDP0000296123, and MDP0000315892/MDP0000317368 in GDR v1.0, respectively) were also mentioned by Trainin et al. (2016). Conversely, the other candidate genes highlighted by these authors were located outside of our largest confidence interval, as were all the candidate genes cited by Celton et al. (2011). Finally, special attention should be given to the MADS-domain containing gene (MD09G1009100 = MDP0000167381 = MDP0000126259 in its shorter version) since it was upregulated in several differential expression situations when comparing the low chilling requirement sport “Castel Gala” with “Royal Gala” (Porto et al., 2015). By contrast, the other two genes (MADS-box: MDP0000207984, and *PRE1*-like: MDP0000320691) highlighted by Porto et al. (2015), were located either on another chromosome or considerably downstream on chromosome 9.

Whilst the candidate genes we identified did not encompass all of those that have previously been proposed to have a role in flowering time, it is clear that a number of them have putative roles in the regulation of flowering time in apple or other plants.

### Putative functions of genes controlling phenotypic variation in apple ripening period

Concerning ripening period, three main genomic regions were identified (on chromosomes 3, 10, and 16) with candidate genes belonging to the NAC family, surrounded by other genes putatively involved in apple ripening. In the genomic region of chromosome 3, two NAC transcription factors (MD03G1222600 and MD03G1222700) are strongly indicated as candidate genes for the control of this trait. A member of this gene family (i.e., ppa008301m, according to the *P. persica* genome version v1.0) was identified in a major locus on chromosome 4 controlling maturity date in peach, and a 9 bp DNA insertion in its last exon was described as a variant putatively linked to early ripening (Pirona et al., 2013). Most interestingly, one of the two NAC genes of apple (i.e., MD03G1222700) cited above showed to be the best homolog of this particular peach NAC gene which was renamed Prupe.4G186800 in the *P. persica* genome version v2.1 (Verde et al., 2017). The second apple NAC gene was also showed to be homolog to the second peach NAC gene cited by Pirona et al. (2013) (i.e., ppa007577m.v1.0 equivalent to Prupe.4G187100.v2.1), and a strong microsynteny was observed between *Malus* and *Prunus* all along the analyzed confidence interval (Table S10). The importance of NAC transcription factors in controlling fruit ripening traits has been described also in tomato (Zhu et al., 2014) and kiwifruit (Nieuwenhuizen et al., 2015). Very recently, two NAC members (called SlNAC4/9) were indicated as regulators of ethylene biosynthesis and ethylene-related genes in tomato (Kou et al., 2016).

The genes identified on chromosome 10 appeared to be involved in the same metabolic pathways: two NAM/NAC (MD10G1288300 and MD10G1299900) and two Apetala2 (MD10G1290400 and MD10G1290900) transcription factors. Members of the plant-specific APETALA2/ethylene response factor (AP2/ERF) superfamily of transcription factors act downstream of the ethylene signaling pathway and are strongly conserved throughout the plant kingdom (Xie et al., 2016). They are apparently associated with several plant developmental and growth processes, including fruit ripening (Licausi et al., 2010; Karlova et al., 2014; Xie et al., 2016).

On chromosome 16, two additional putative transcription factors encoding either a NAM/NAC domain (MD16G1125800) or a TIFY domain (MD16G1127400) were identified as well as one gene encoding an auxin responsive protein (MD16G1124300) and one gene for a sugar bidirectional transporter (MD16G1125300) with high homology with a senescence associated protein (SAG 29) of *Arabidopsis*. TIFY transcription factors comprise a plant-specific family involved in the regulation of various developmental processes and responses to phytohormones. Among the 30 members of this family characterized in apple (Li et al., 2015), are the jasmonate zim-domain (JAZ) proteins, known to be repressors of JA signaling and, consequently, actors of the cross-talk among multiple hormone signaling pathways including ethylene and gibberellins (An et al., 2016). The expression patterns of genes in the JA biosynthesis pathway was found to be correlated with genes in the ethylene biosynthesis pathway, emphasizing the role of JA biosynthesis and its signaling on apple fruit maturation (Lv et al., 2015).

Altogether, candidate genes identified after GWAS highlight the probable role of transcription factors, controlling the ethylene biosynthesis or regulatory pathway, for ripening in apple. Other candidates such as the gene encoding for an auxin responsive protein may also be considered since an ethylene–auxin interplay at a late ripening stage has been proposed in apple (Tadiello et al., 2016).

## Future perspectives

GWAS mapping is a powerful tool for the identification of genomic regions associated with important traits, but results can be restricted by too much genetic heterogeneity, insufficient marker density and an overly strong impact of population structure. In the present study, narrow genomic regions controlling two phenological traits and a rather low number of candidate genes were identified, while other regions remained unidentified because of the relationship between traits and geographic structure. Enlarging the diversity panel with more genotypes, especially from Southern and Northern+Eastern groups, might improve detection of loci associated with those traits in each geographic group. Also, a combination of linkage and association analyses may achieve higher statistical power and resolution (Jansen et al., 2003; Flint-Garcia et al., 2005; Pascual et al., 2016) and reduce the confidence interval of the detected genomic regions which would call for validating the function of certain candidate genes by genetic transformation, especially gene editing (Busov et al., 2005; Nishitani et al., 2016). Still, the current set of phenotypic and genotypic data is already useful to establish genome-wide predictions (Meuwissen et al., 2001; Muranty et al., 2015) of the breeding values of the studied genotypes for these two traits.

## Author contributions

JU, HM, and DL carried out the statistical analyses under the supervision of C-ED and ST. CD and ER managed the leaf sample collection and part of DNA extraction. AT performed the remaining DNA extraction and the whole runs of the Axiom® plates on the GeneTitan® under the supervision of CP. CD performed the whole SNP genotyping analysis and validation process with the help of HM and C-ED. Selection of germplasm and acquiring phenotypic data were performed by C-ED, AG, RG, LF, ML, PH, MO, FP, JS, HN, RGr, LD, and ST. MB and SM brought expertise about GWAS methodology and results interpretation. SA performed the candidate gene analysis thanks to the new apple genome sequence developed by ND and J-MC. C-ED conceived and coordinated the study. FL coordinated the EU FruitBreedomics project including this study. JU, HM, and C-ED wrote the manuscript with decisive contributions of SA and LD. HN, MO, MT, LB, RV, MB, LG-G, and SM critically reviewed the manuscript. All authors read and approved the final manuscript.

### Conflict of interest statement

The authors declare that the research was conducted in the absence of any commercial or financial relationships that could be construed as a potential conflict of interest. The reviewer AMA and handling editor declared their shared affiliation.

## Abbreviations

CRA-W: Centre Wallon de Recherche Agronomique [Gembloux (Belgium)]
EBIC: Extended Bayesian Information Criterion
GDDH13 genome: version (v1.1) released for the apple genome based on the doubled haploid GDDH13
GWAS: Genome-Wide Association Study
INRA: Institut National de la Recherche Agronomique [Angers (France]
LD: linkage disequilibrium
MLMM: multi-locus mixed model
NFC: University of Reading [Brogdale (United Kingdom)]
PCA: Principal Component Analysis
QTL: Quantitative Trait Loci
RBIPH: Research and Breeding Institute of Pomology Holovousy [Holovousy (Czech Republic)]
SLU: Swedish University of Agricultural Sciences [Alnarp (Sweden)]
SNP: Single Nucleotide Polymorphism
UNIBO: University of Bologna [Bologna (Italy)].
