# Supplementary material for: Genome-Wide Association Mapping of Flowering and Ripening Periods in Apple
Source: Front Plant Sci. 2017 Nov 10;8:1923. doi: 10.3389/fpls.2017.01923 (PMC5686452; doi:10.3389/fpls.2017.01923)
Supplement: Supplementary file 1 [file Image1.PDF]

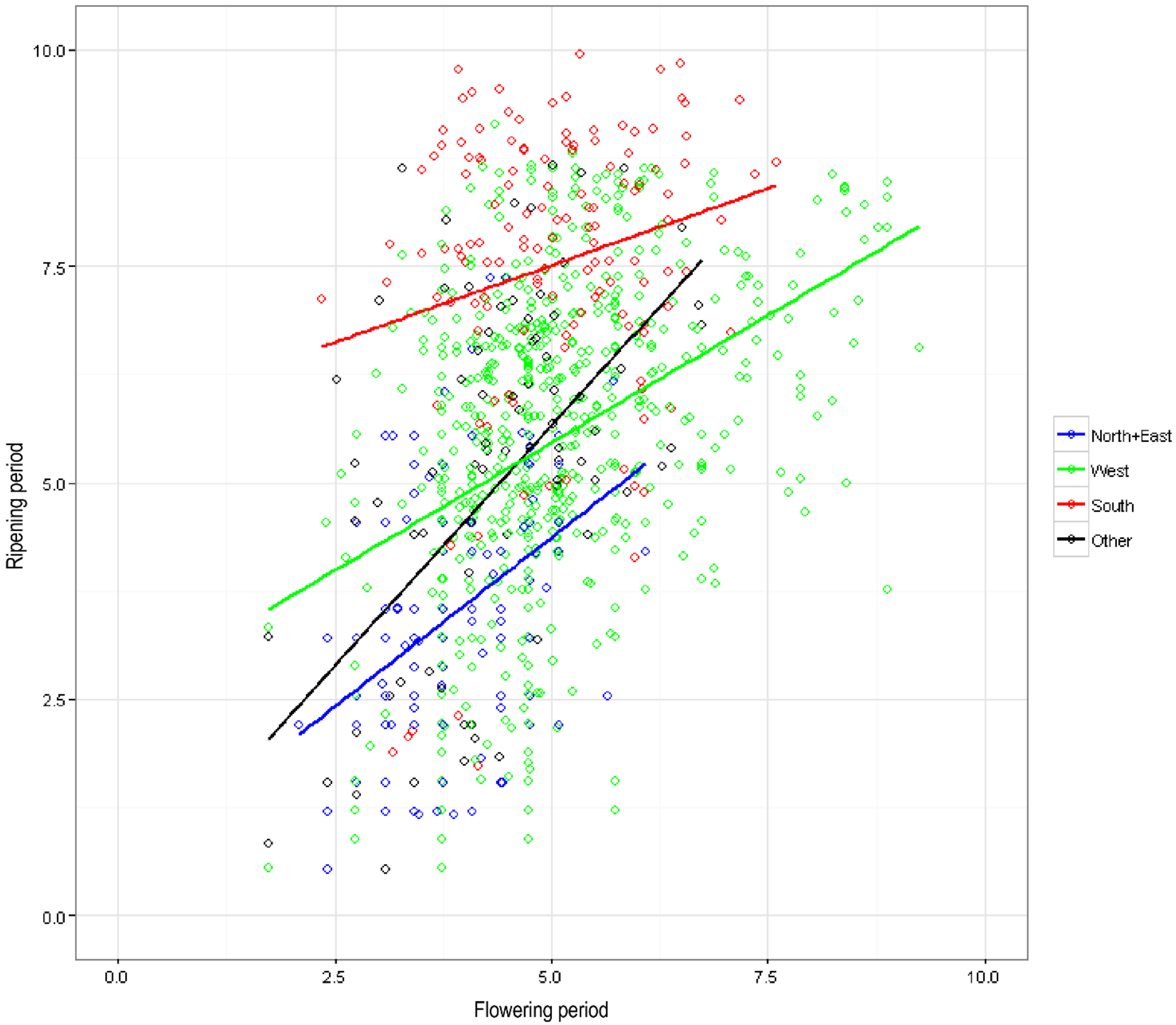

Figure S1. Graph of the genotypic means of the genotypes for flowering period versus ripening period according to the three geographic groups. The geographic groups are depicted using the following color codes: Blue = North+East group; Green = West group; Red = South group; Black = Other. Linear regressions lines are indicated for the three groups with the corresponding colors.
