# Supplementary material for: Genome-Wide Association Mapping of Flowering and Ripening Periods in Apple
Source: Front Plant Sci. 2017 Nov 10;8:1923. doi: 10.3389/fpls.2017.01923 (PMC5686452; doi:10.3389/fpls.2017.01923)
Supplement: Supplementary file 2 [file Image2.PDF]

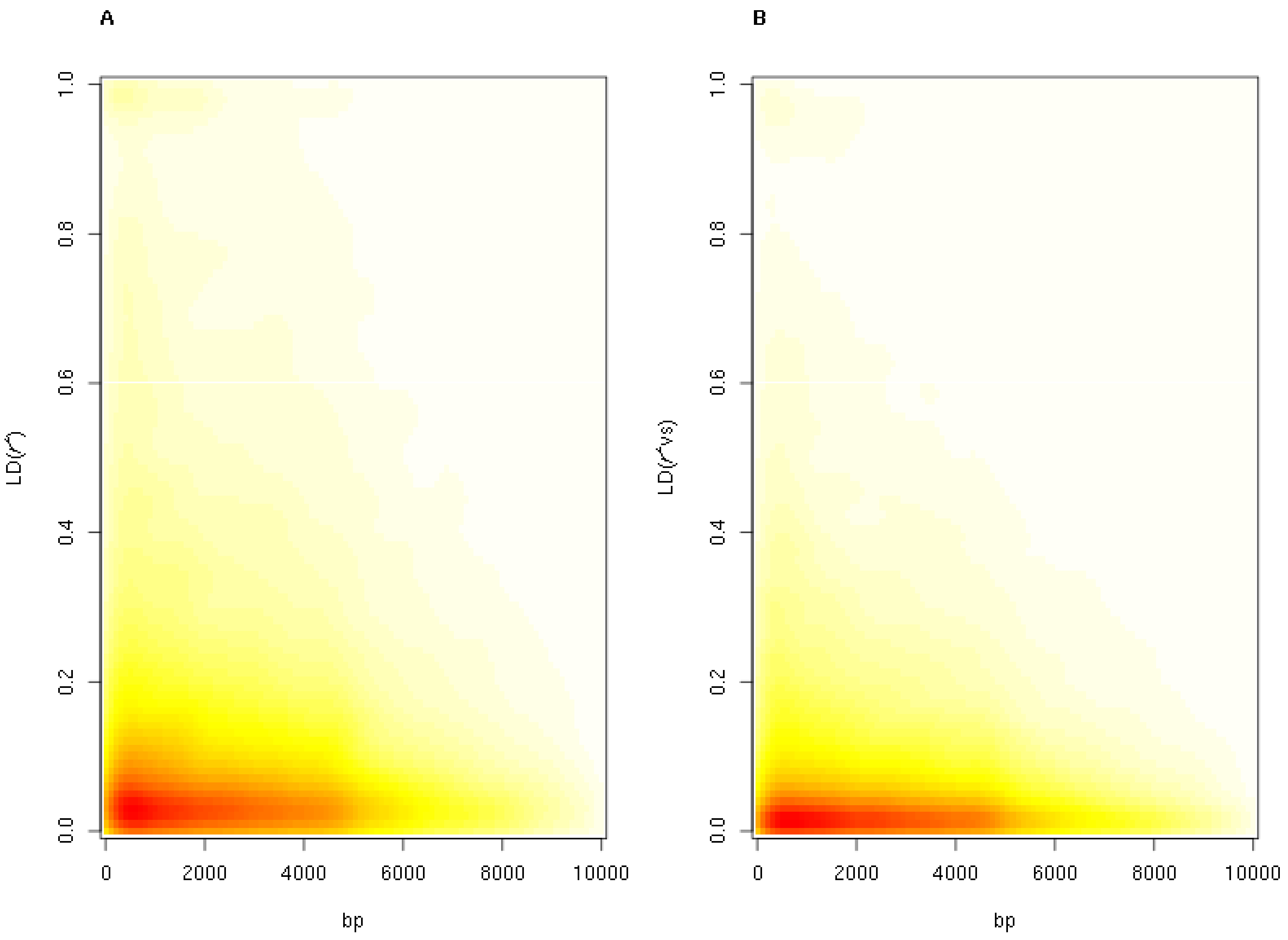

Figure S2. LD density plot based on the usual r^2 (A) and after correcting for relatedness and population structure (r^2_{vs}) (B).
