# Supplementary material for: Genome-Wide Association Mapping of Flowering and Ripening Periods in Apple
Source: Front Plant Sci. 2017 Nov 10;8:1923. doi: 10.3389/fpls.2017.01923 (PMC5686452; doi:10.3389/fpls.2017.01923)

Figure S4. Manhattan plots for ripening period in the whole population (A) based on a single-locus mixed model (upper part) and on a multi-locus mixed model (lower part) at a Bonferroni-corrected threshold of 0.05 (dashed horizontal line). Manhattan plots for ripening period in each of the six collections (B - G) based on a single-locus mixed model (upper part) and on a multi-locus mixed model (lower part) at a Bonferroni-corrected threshold of 0.05 (dashed horizontal line): INRA (B), UNIBO (C), CRA-W (D), RBIPH (E), NFC (F) and SLU (G). The fictive chromosome 0 contained the SNPs in all unassigned scaffolds and the fictive chromosome 19 contained SNPs with no position after blasting on the GDDH13 genome.

A whole population single-locus mixed model

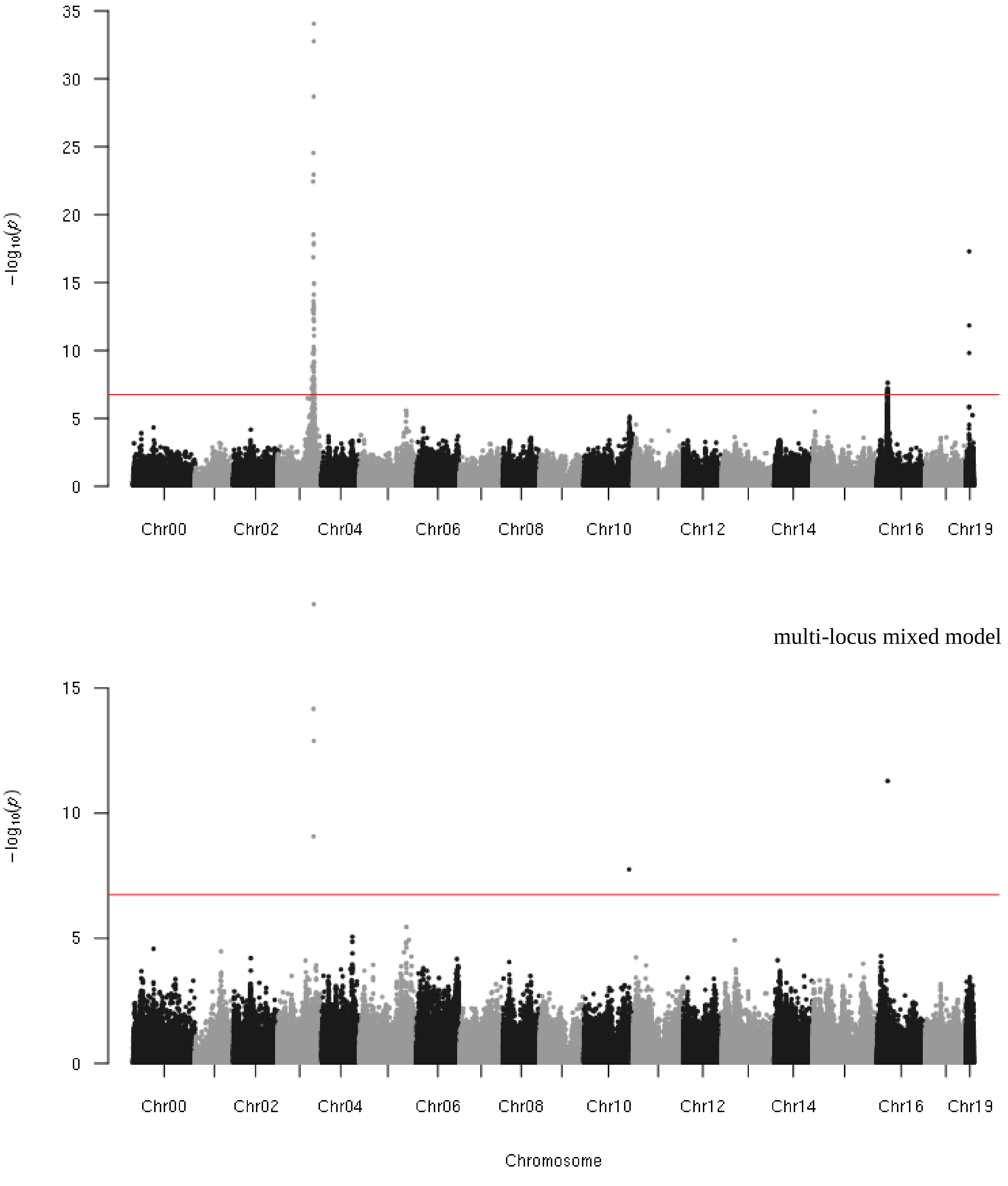

Continued

B INRA single-locus mixed model multi-locus mixed model

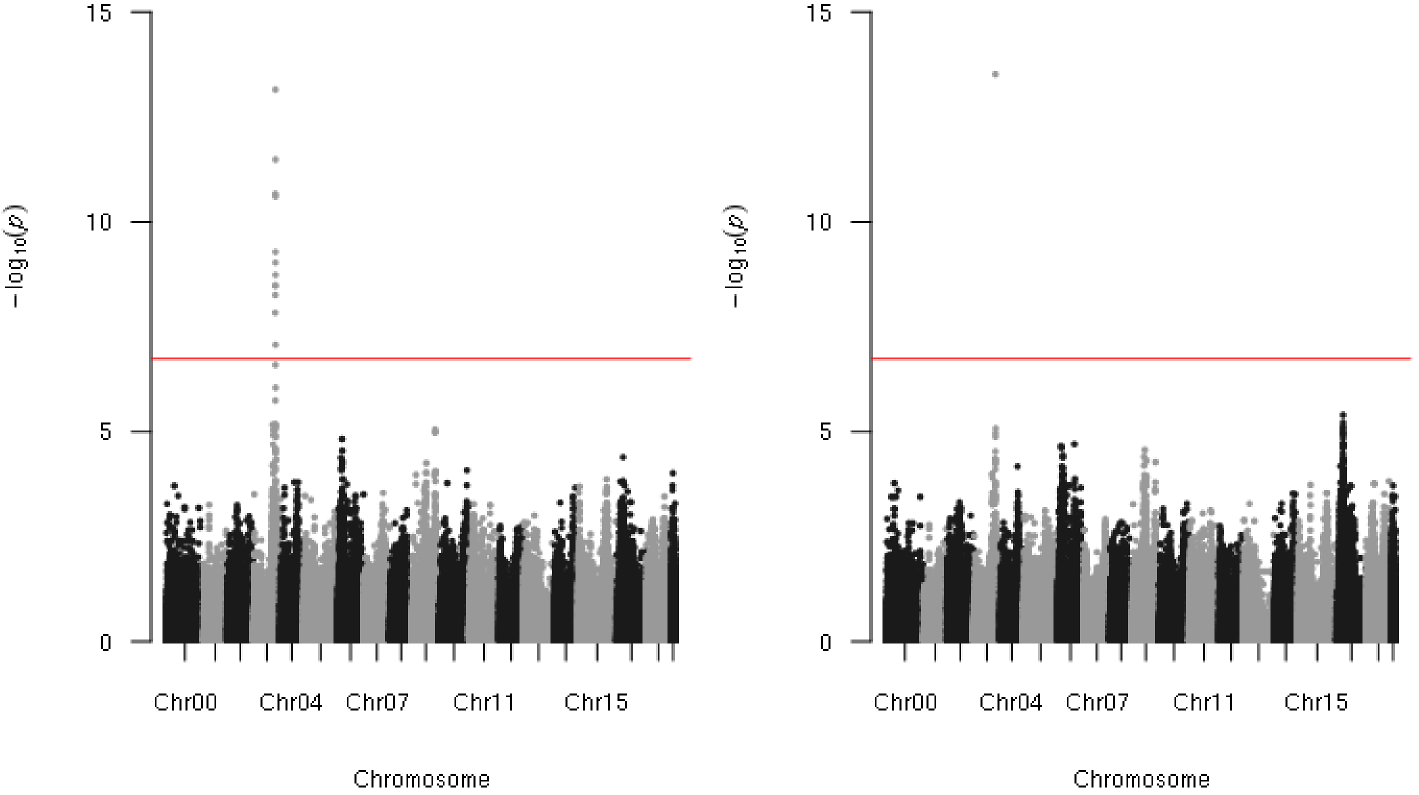

C UNIBO single-locus mixed model multi-locus mixed model

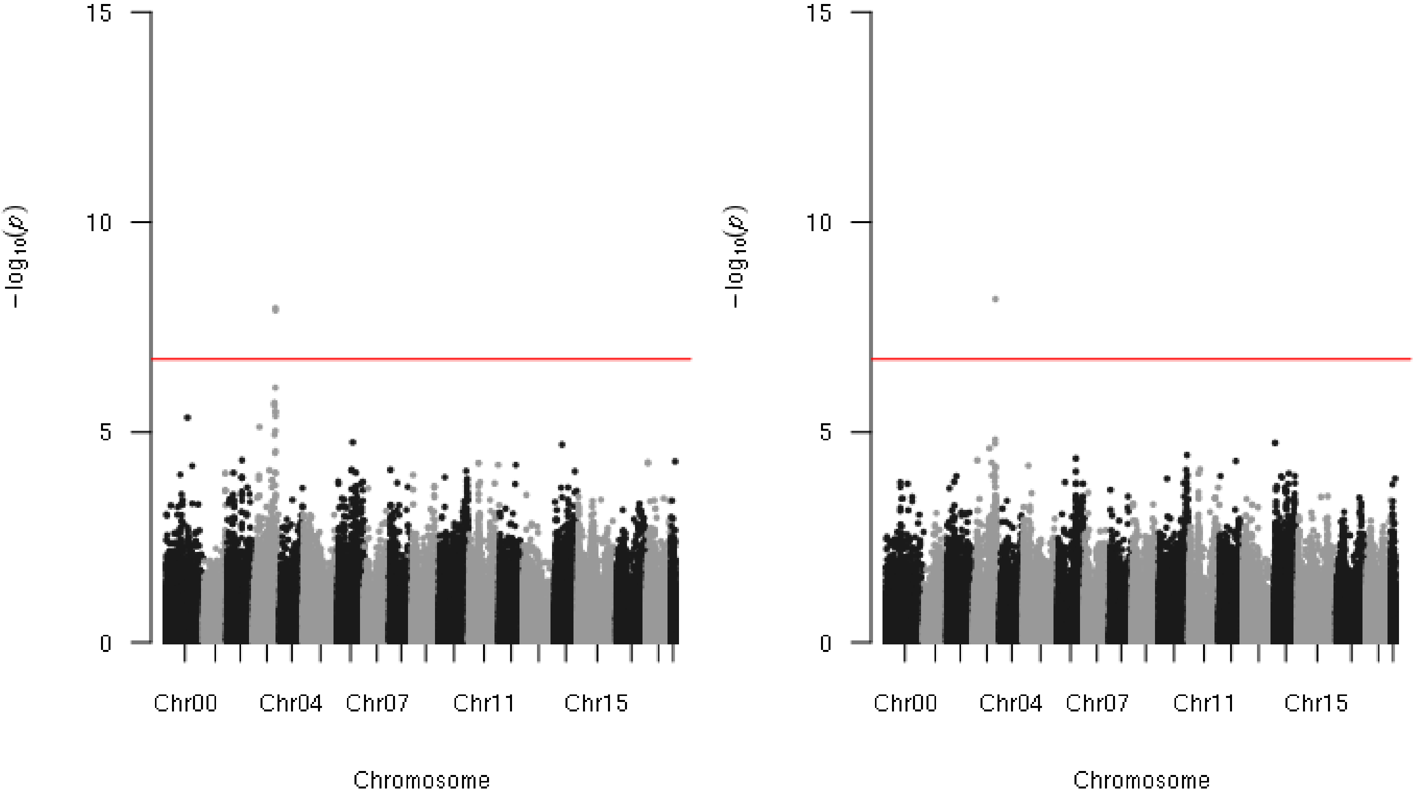

Continued

D CRA-W single-locus mixed model multi-locus mixed model

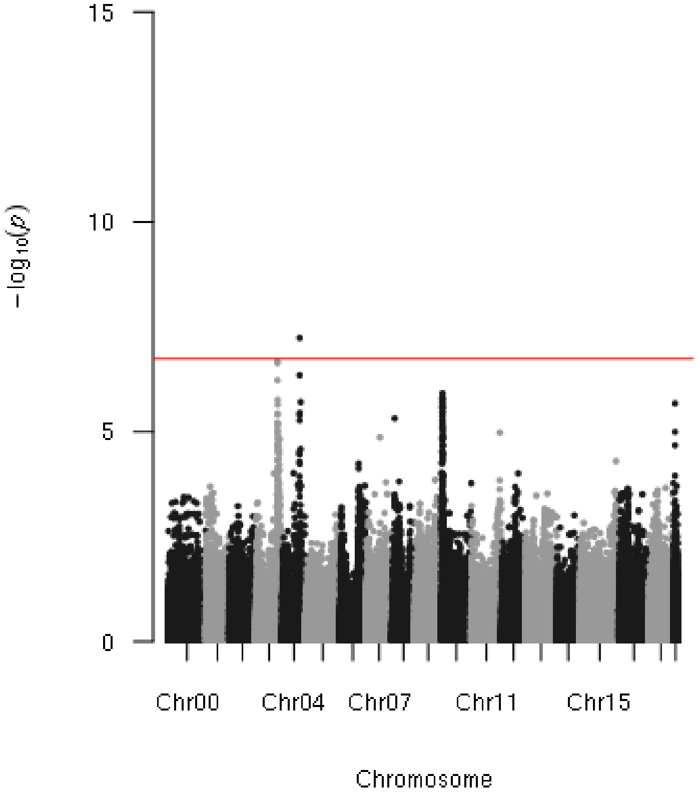

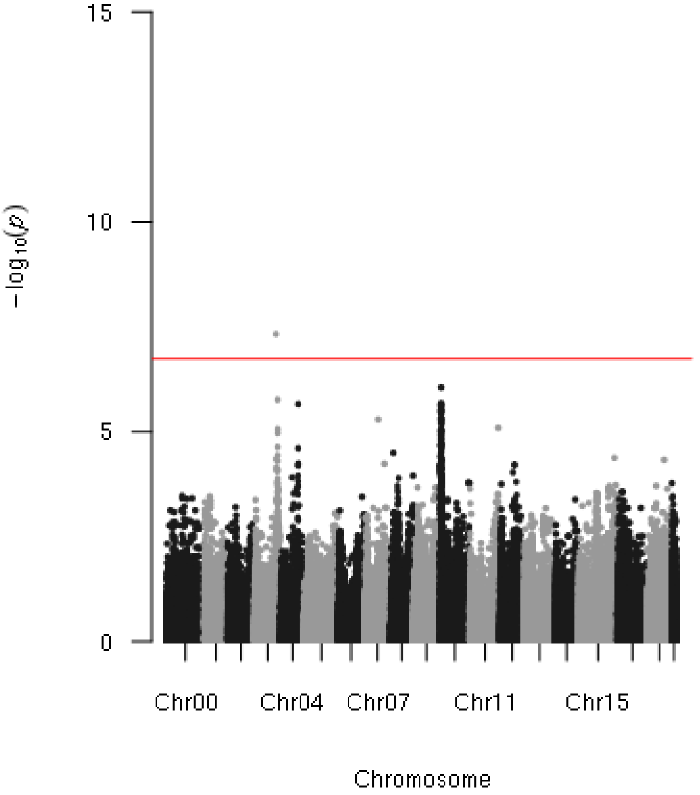

E RBIPH single-locus mixed model multi-locus mixed model

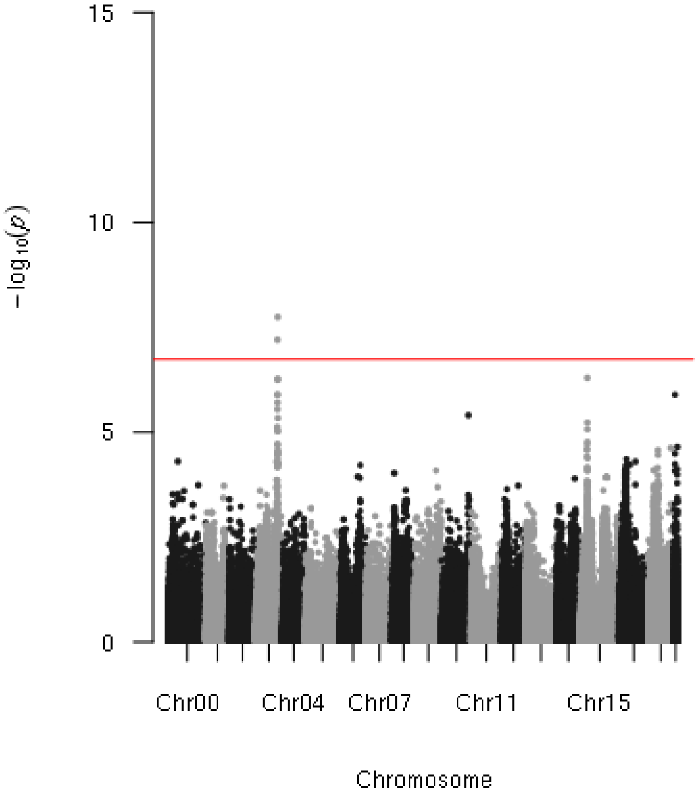

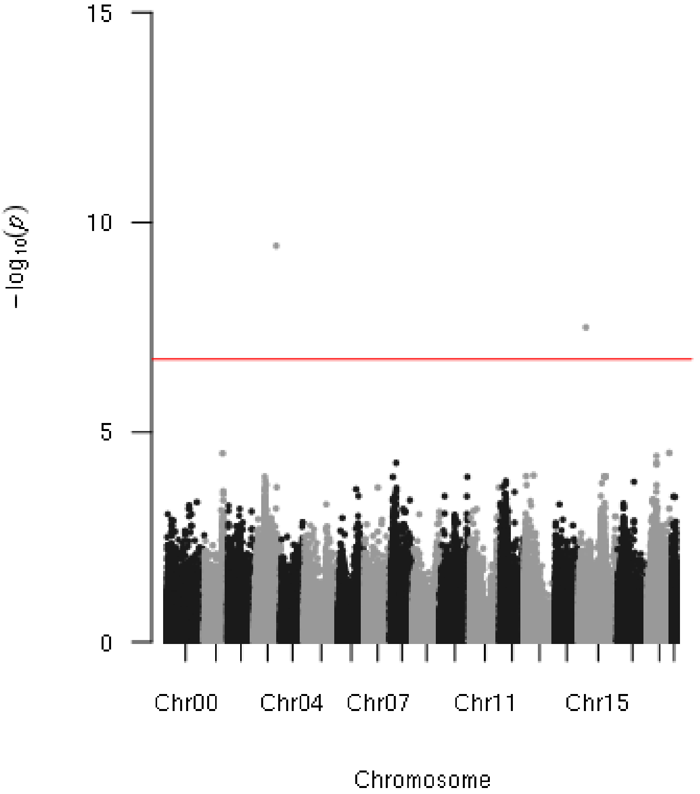

Continued

F NFC single-locus mixed model multi-locus mixed model

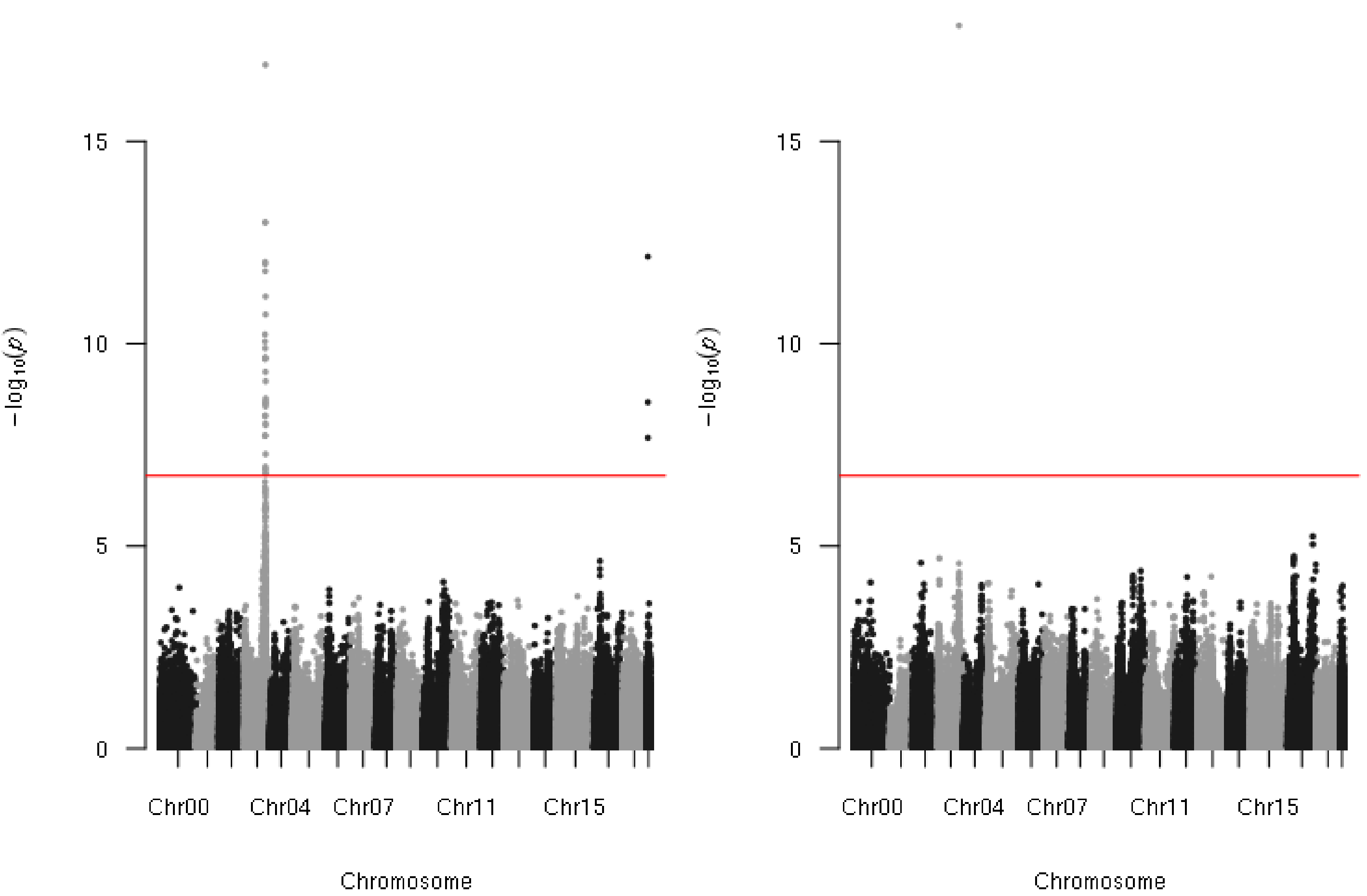

G SLU single-locus mixed model multi-locus mixed model

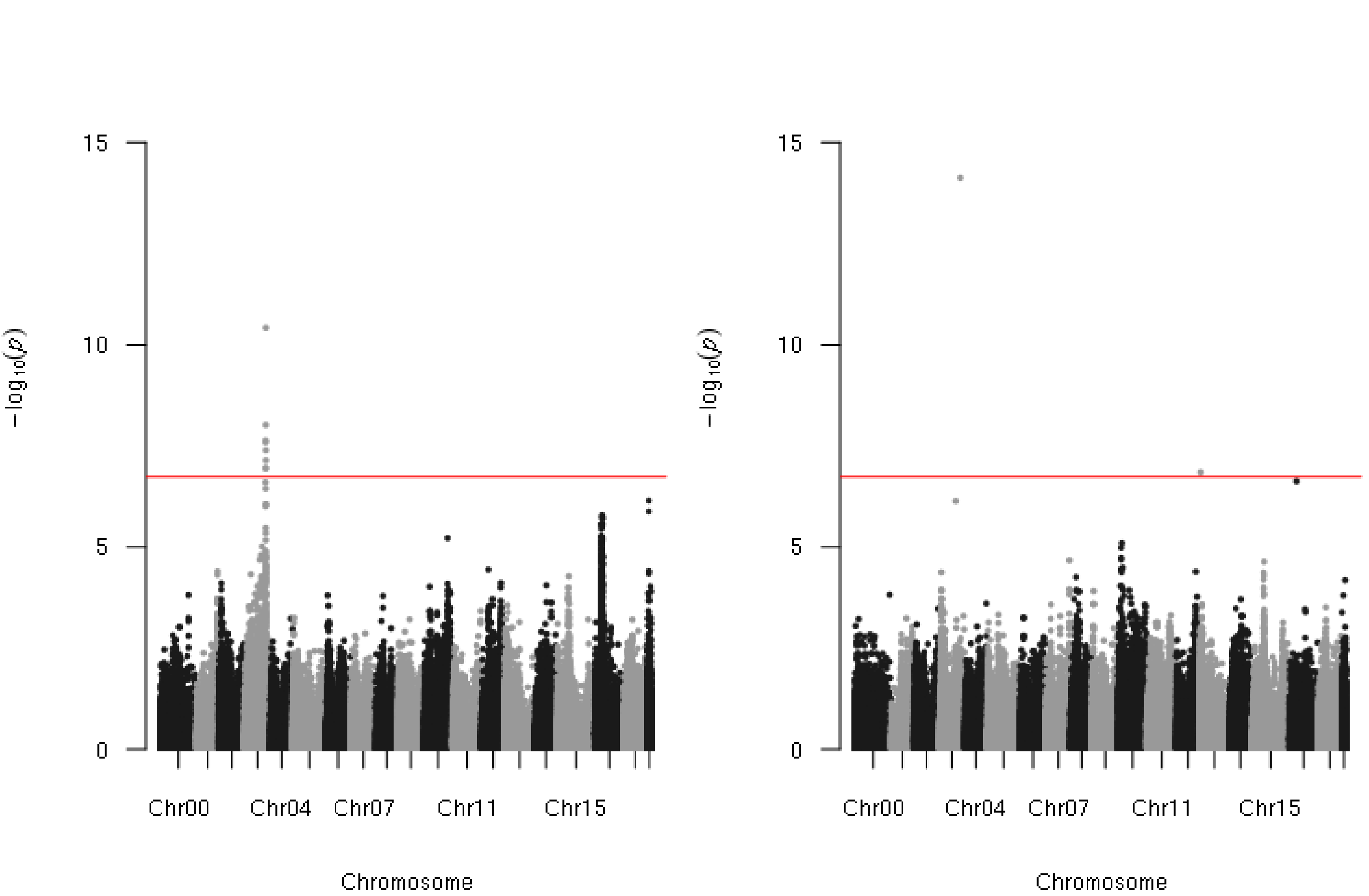

Supplement: Supplementary file 4 [file Image4.PDF]
