# Supplementary material for: Genome-Wide Association Mapping of Flowering and Ripening Periods in Apple
Source: Front Plant Sci. 2017 Nov 10;8:1923. doi: 10.3389/fpls.2017.01923 (PMC5686452; doi:10.3389/fpls.2017.01923)
Supplement: Supplementary file 5 [file Image5.PDF]

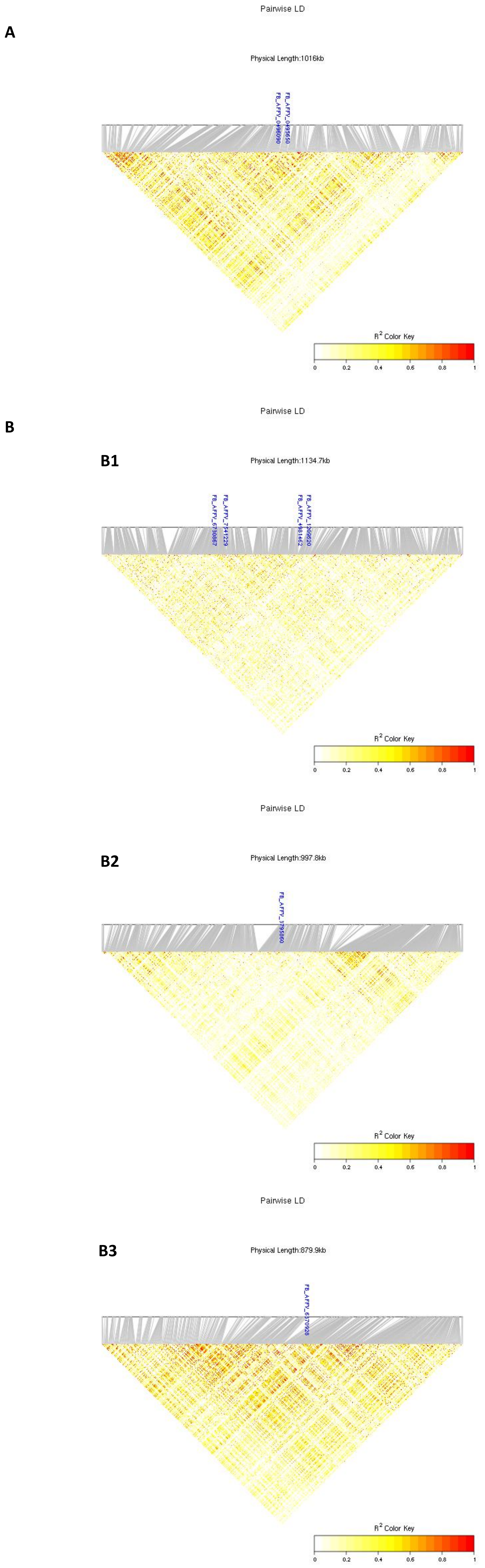

Figure S5. Heat map of linkage disequilibrium for the region of ~1Mb surrounding the two SNPs selected as cofactors located on chromosome 9 in the GWA analysis of the whole population for flowering period (A). Heat maps of linkage disequilibrium for the regions of ~1Mb surrounding the six SNPs selected as cofactors in the GWA analysis of the whole population for ripening period (B): the surrounding regions of the four SNPs selected as cofactors located on chromosome 3, of the SNP selected as cofactor on chromosome 10, and of the SNP selected as cofactor on chromosome 16 are shown in B1, B2 and B3, respectively. Individual data points reflect usual r^2 for all possible pairs of SNP markers located in the above mentioned regions. Positions of the SNPs selected as cofactors in the GWA analyses are approximately indicated by blue letters.
