# Supplementary material for: Genome-Wide Association Mapping of Flowering and Ripening Periods in Apple
Source: Front Plant Sci. 2017 Nov 10;8:1923. doi: 10.3389/fpls.2017.01923 (PMC5686452; doi:10.3389/fpls.2017.01923)
Supplement: Supplementary file 6 [file Image6.PDF]

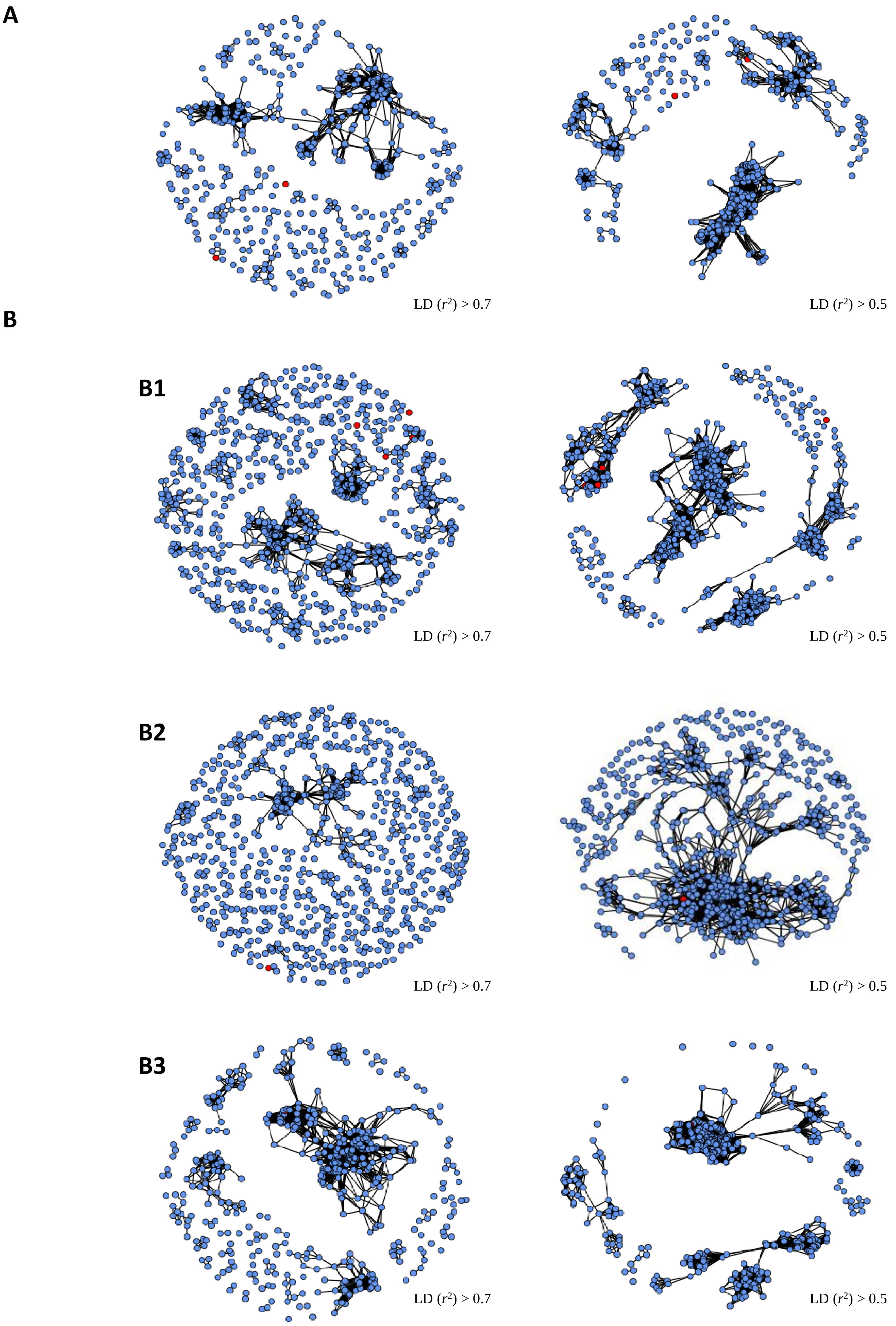

Figure S6. A. Network plots showing the LD-interconnections among SNPs at r^2 higher than 0.7 and 0.5 in the surrounding regions of ~1Mb of the two SNPs selected as cofactors located on chromosome 9 for flowering period in the GWA analysis of the whole population. The two SNPs selected as cofactors are indicated by red dots. B. Network plots showing the LD-interconnections among SNPs at r^2 higher than 0.7 and 0.5 in the surrounding regions of ~1Mb of the six SNPs selected as cofactors for flowering ripening period in the GWA analysis of the whole population. The four SNPs selected as cofactors and located on chromosome 3 are indicated by red dots in B1. The SNP selected as cofactor and located on chromosome 10 is indicated by a red dot in B2. The SNP selected as cofactor and located on chromosome 16 is located within the biggest cluster of blue dots in B3.
