# Supplementary material for: Genome-Wide Association Mapping of Flowering and Ripening Periods in Apple
Source: Front Plant Sci. 2017 Nov 10;8:1923. doi: 10.3389/fpls.2017.01923 (PMC5686452; doi:10.3389/fpls.2017.01923)

Figure S7. Box plots representing the allelic effects of the two SNPs selected in the GWA analysis for flowering period in the whole population. Allelic effects for the whole population and the three geographic groups (i.e., North+East, West and South) are provided. Box plots show the median (middle line), the 25% and 75% quartiles (hinges) and the outer value within the 1.5 × interquartile range (whiskers). Allelic effects at SNP.9-1 and SNP.9-2 are shown in A and B, respectively. Each dot represents one genotype.

A SNP.9-1

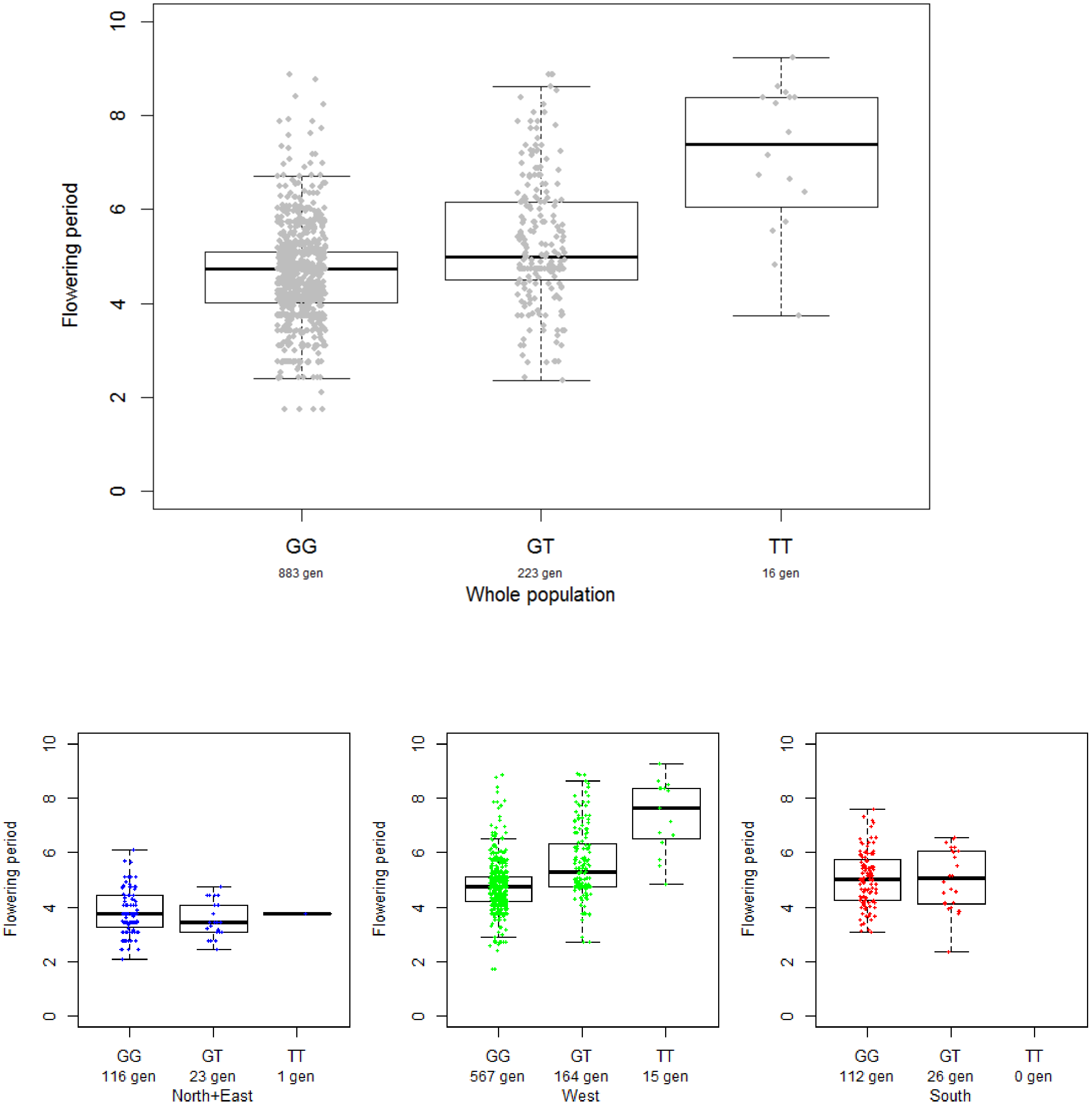

Continued

B SNP.9-2

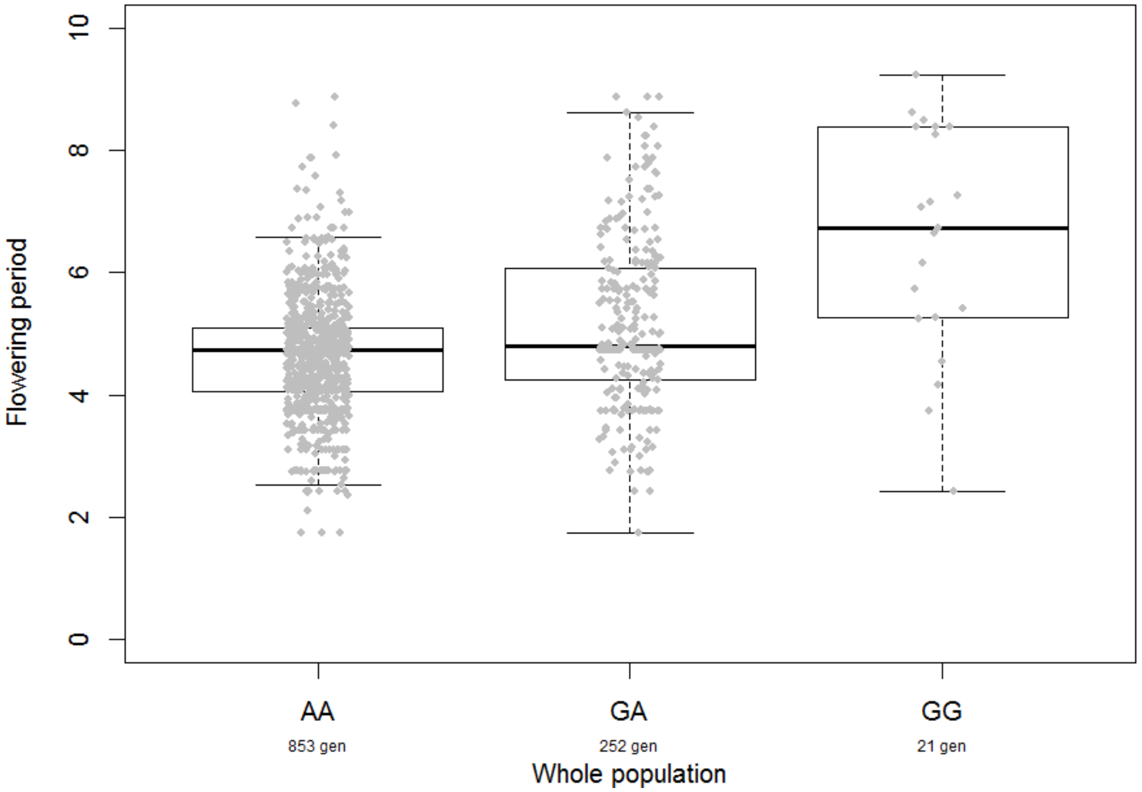

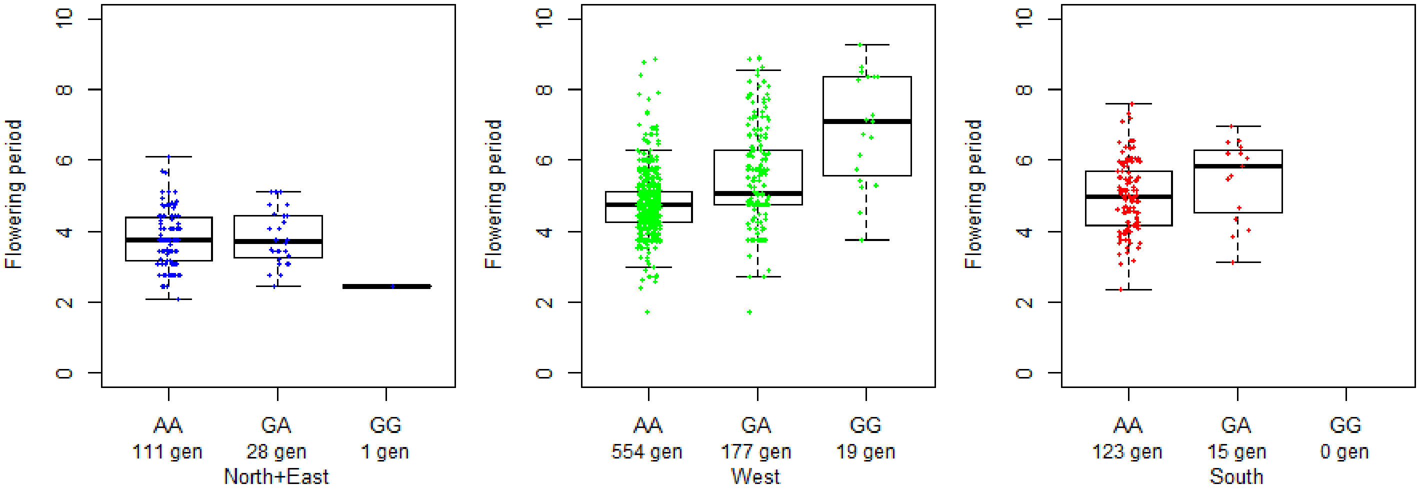

Supplement: Supplementary file 7 [file Image7.PDF]
