# Supplementary material for: Genome-Wide Association Mapping of Flowering and Ripening Periods in Apple
Source: Front Plant Sci. 2017 Nov 10;8:1923. doi: 10.3389/fpls.2017.01923 (PMC5686452; doi:10.3389/fpls.2017.01923)

Figure S8. Box plots representing the allelic effects of the six SNPs selected in the GWA analysis for ripening period in the whole population. Allelic effects for the whole population and the three geographic groups (i.e., North+East, West and South) are provided. Box plots show the median (middle line), the 25% and 75% quartiles (hinges) and the outer value within the 1.5 × interquartile range (whiskers). Allelic effects at SNP.3-3 are shown in A, at SNP.3-4 in B, at SNP.3-6 in C, at SNP.3-7 in D, at SNP.10-1 in E and at SNP.16-1 in F. Each dot represents one genotype.

A SNP.3-3

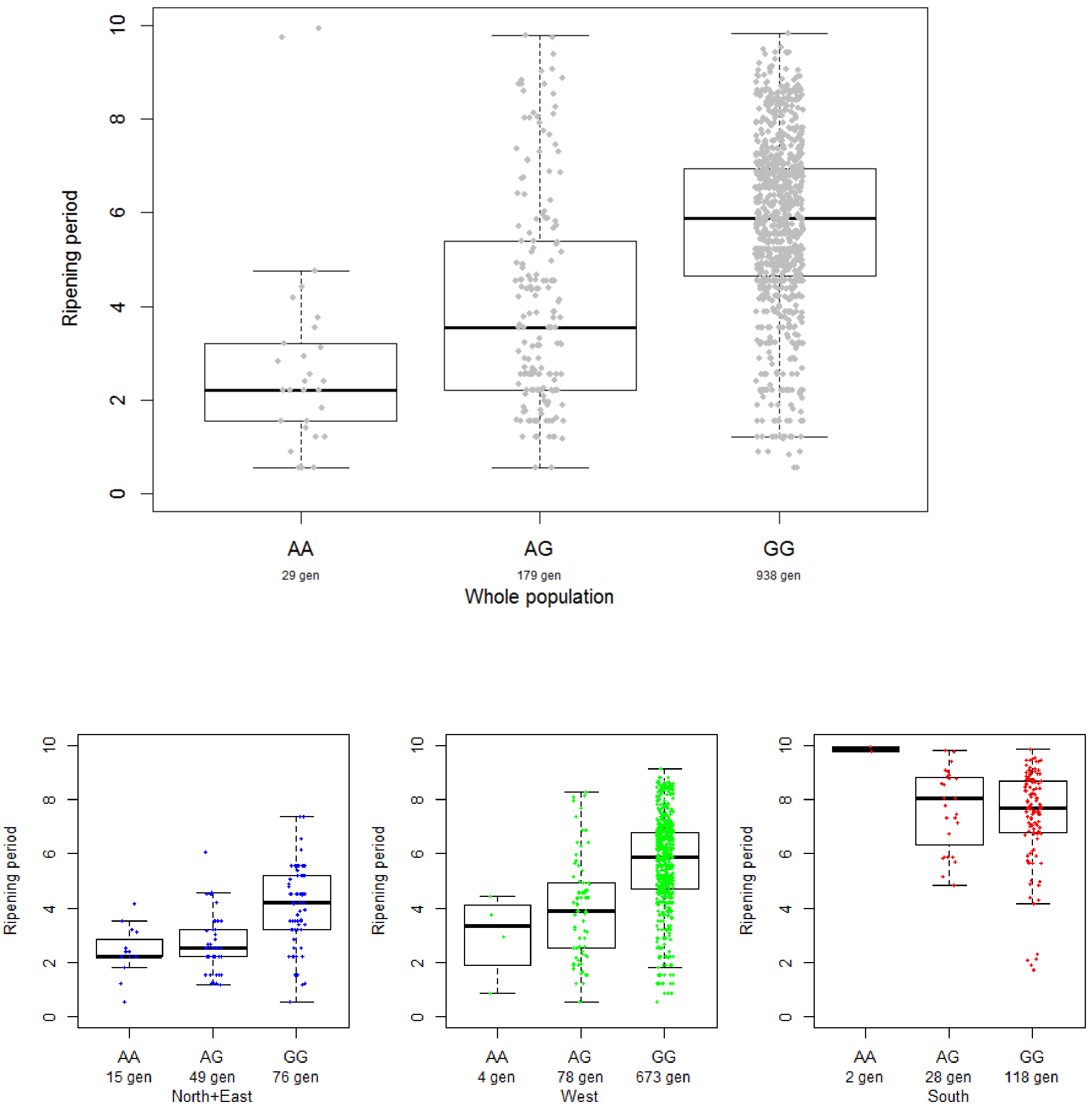

Continued

B SNP.3-4

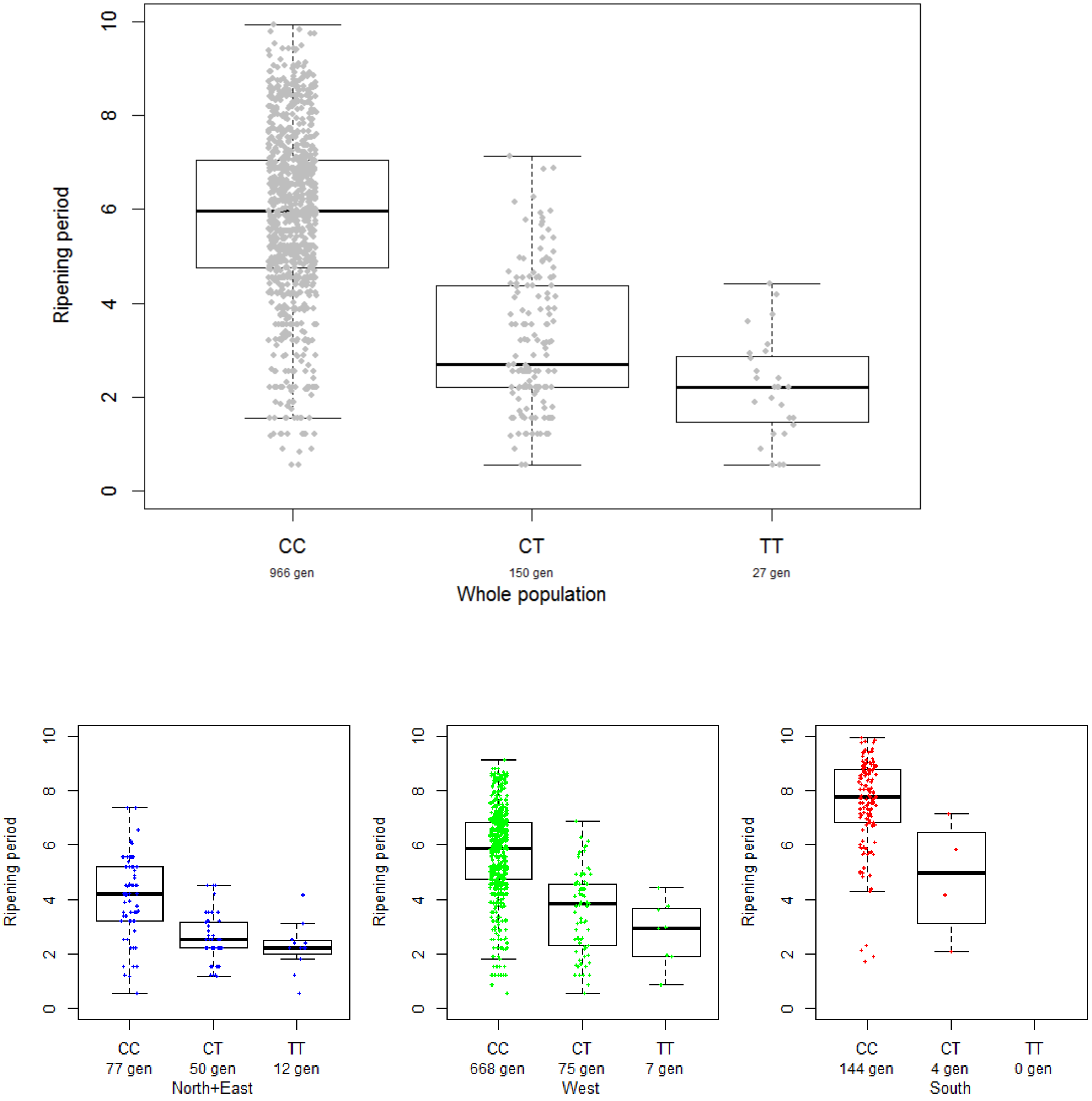

Continued

C SNP.3-6

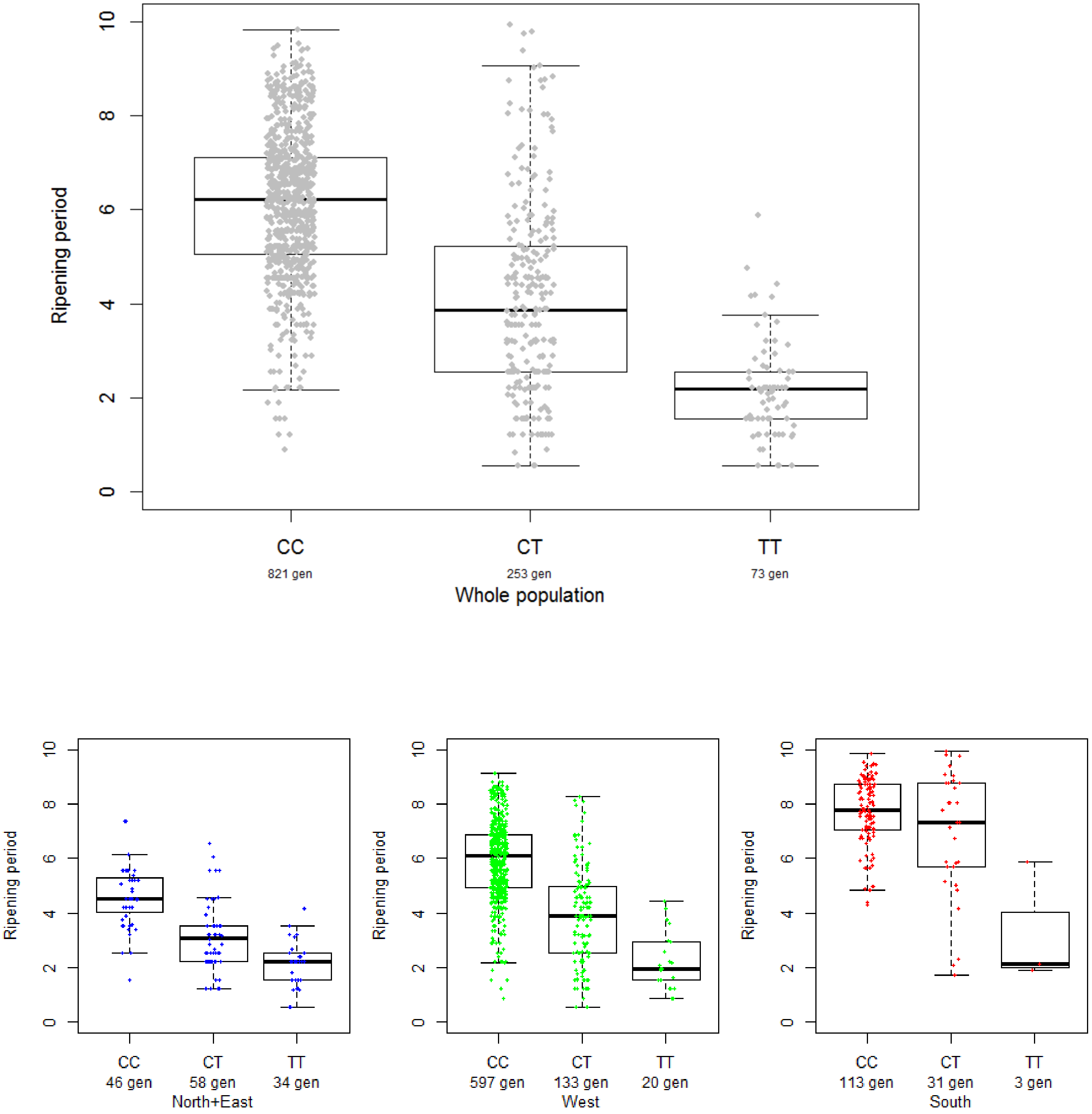

Continued

D SNP.3-7

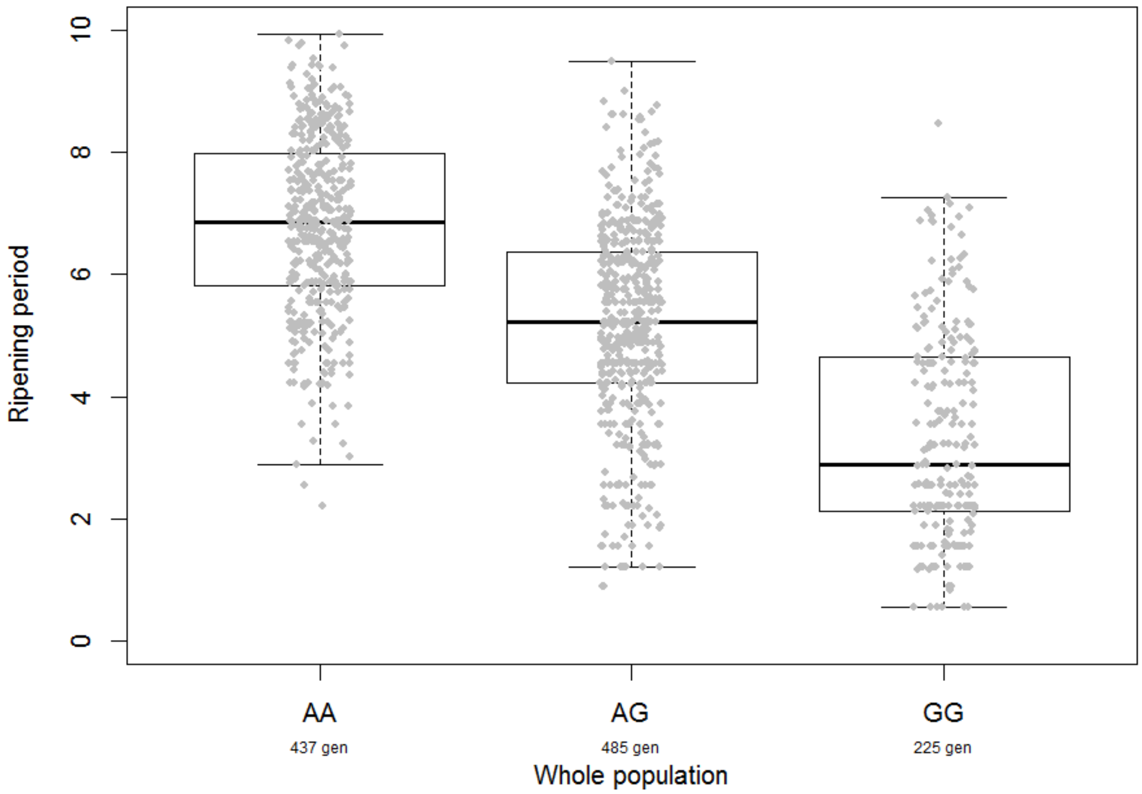

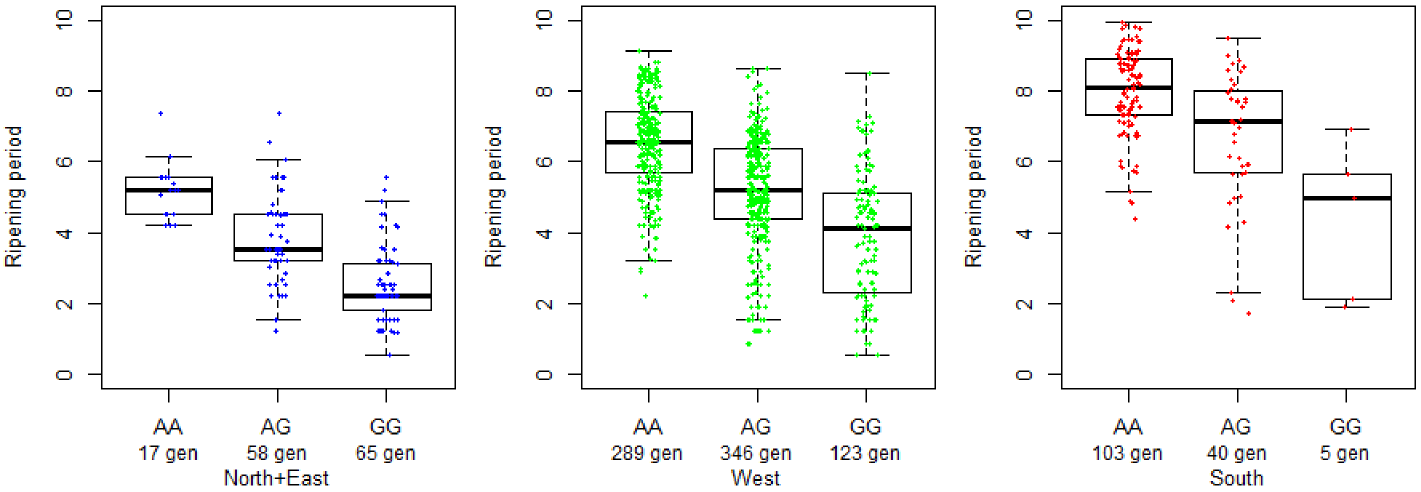

Continued

E. SNP.10-1

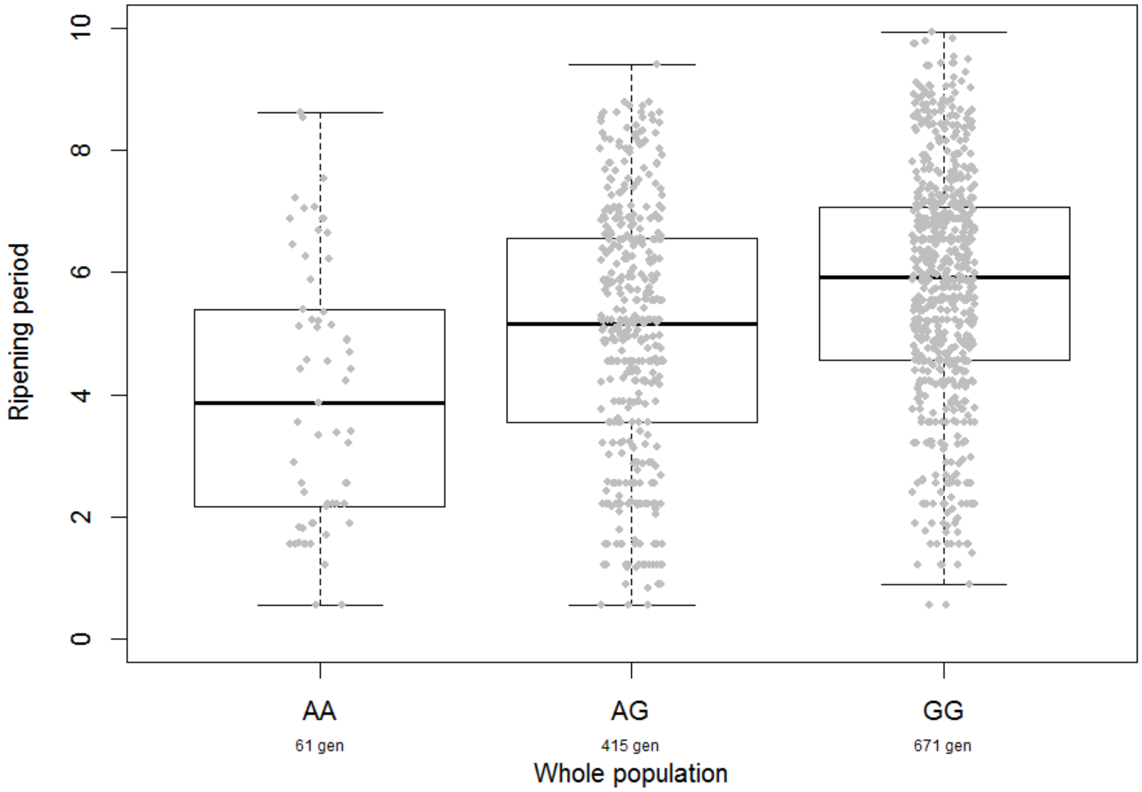

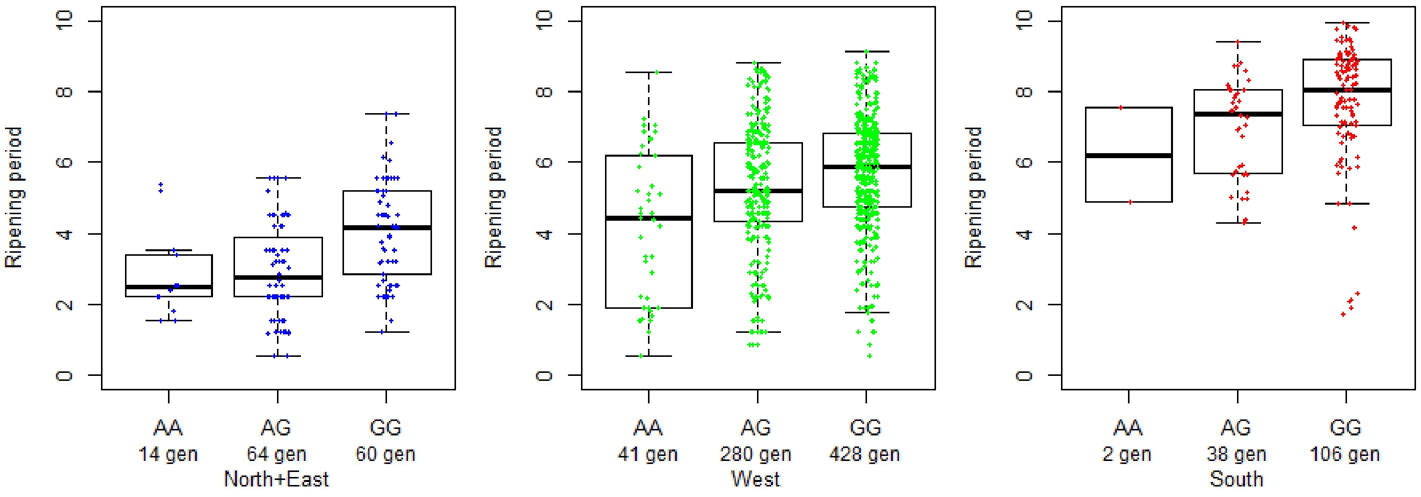

Continued

F. SNP.16-1

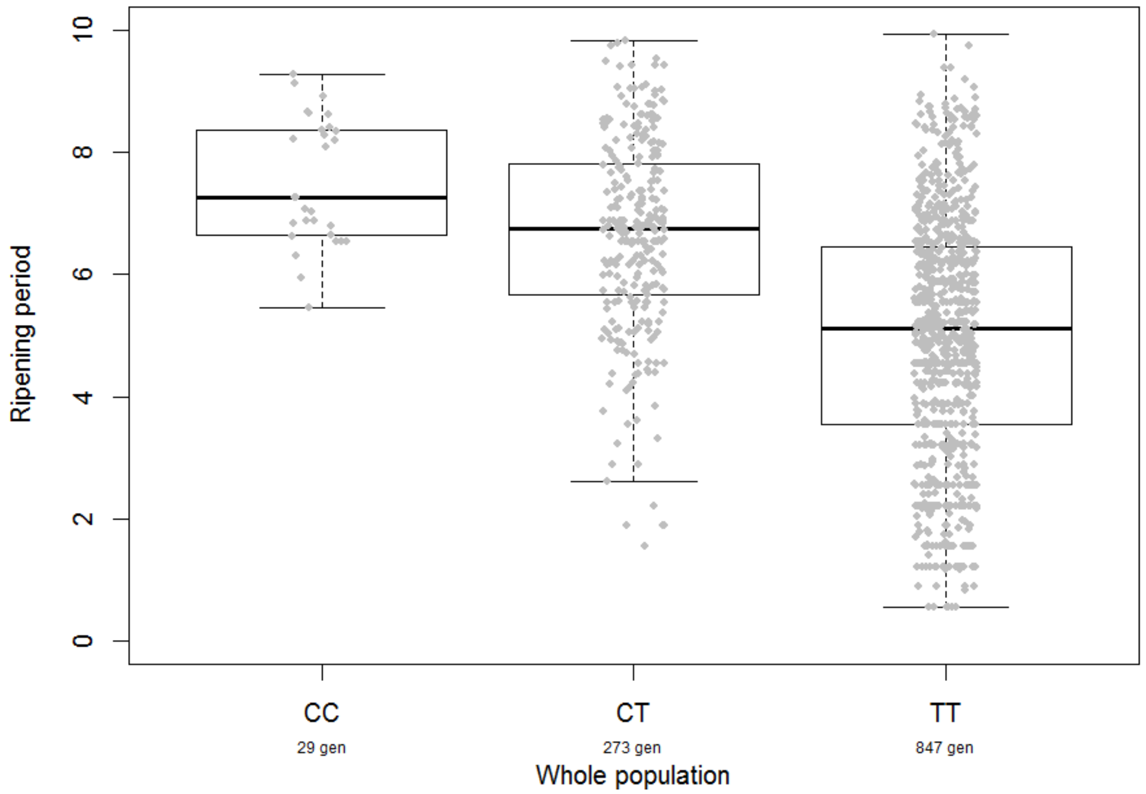

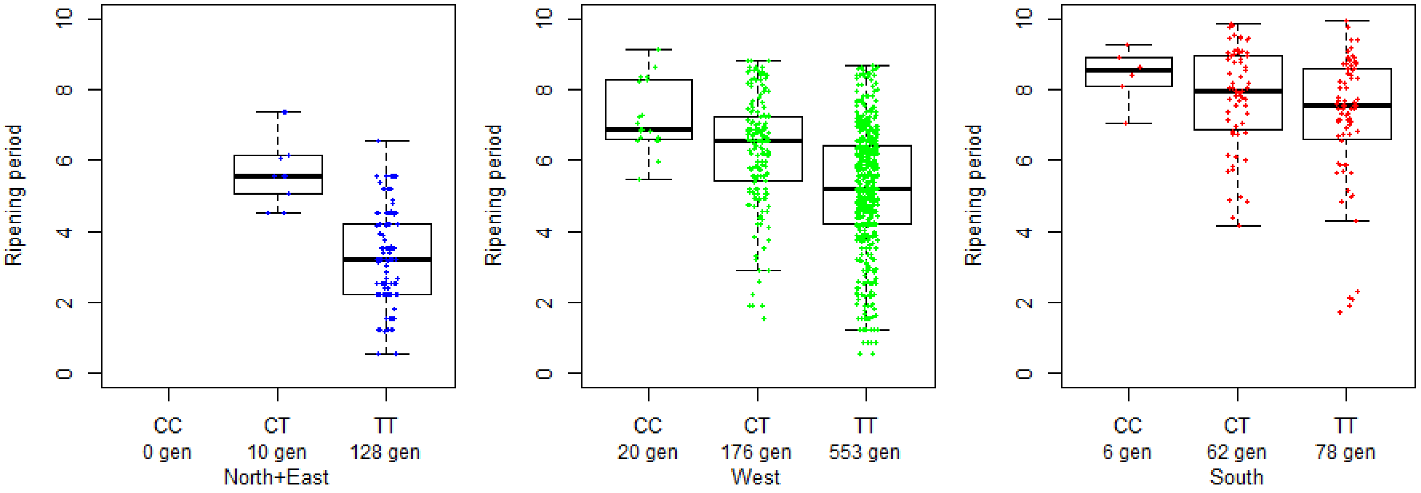

Supplement: Supplementary file 8 [file Image8.PDF]
